# *CXCL13* as a Prognostic Biomarker and Immune Microenvironment-Associated Gene in Endometrial Carcinoma: A Multi-Omics Investigation

**DOI:** 10.3390/biology15130987

**Published:** 2026-06-23

**Authors:** Yiwen Sun, Xiaoyv Wang, Fangzheng Wu, Yanglin Ji, Jun Xie

**Affiliations:** The School of Life Science, Fudan University, 2005 Songhu Road, Shanghai 200438, China; 23210700050@m.fudan.edu.cn (Y.S.);

**Keywords:** *CXCL13*, tumor microenvironment, immune infiltration, endometrial cancer, TCGA multi-omics

## Abstract

Immune remodeling within the tumor microenvironment (TME) influences the progression and clinical outcome of uterine corpus endometrial carcinoma (UCEC), but the contribution of chemokine-related regulatory genes remains incompletely characterized. This study aimed to evaluate the prognostic relevance of *CXCL13* and its association with immune microenvironmental features in UCEC using publicly available transcriptomic and single-cell datasets. RNA-sequencing profiles and clinical annotations from 589 UCEC cases in The Cancer Genome Atlas (TCGA) were analyzed to assess TME composition using ESTIMATE (Estimation of Stromal and Immune cells in MAlignant Tumours using Expression data) and CIBERSORT (Cell-type Identification By Estimating Relative Subsets Of RNA Transcripts), followed by survival analysis, differential gene expression analysis, protein–protein interaction network construction, Cox regression, and gene set enrichment analysis. A public single-cell RNA-sequencing dataset from the Gene Expression Omnibus (GEO; GSE173682) was further used to infer the cellular sources of *CXCL13*. Elevated *CXCL13* expression was associated with favorable overall survival and enrichment of immune-activation pathways. CIBERSORT-based analysis indicated that high *CXCL13* expression correlated with increased estimated fractions of CD8^+^ T cells and plasma cells, together with transcriptional features related to tertiary lymphoid structure-associated immune activation, whereas several immunosuppressive cell populations showed lower estimated abundance. Single-cell analysis suggested that *CXCL13* was mainly expressed by follicular helper T cells and exhausted CD8^+^ T cells. These findings indicate that *CXCL13* may serve as a prognostic biomarker associated with an immune-active TME in UCEC. Further histological, spatial, and functional validation is warranted to confirm its mechanistic role and translational potential.

## Simple Summary

Endometrial cancer is a common cancer of the female reproductive system, and its incidence is rising worldwide. How immune cells interact with tumors strongly influences patient outcomes. In this study, we analyzed public genetic data to determine whether a molecule called *CXCL13* is linked to survival and immune activity in endometrial cancer. We found that patients with higher *CXCL13* levels lived longer and showed more signs of active immune cells—such as T cells and antibody-producing cells—within their tumors. Using single-cell analysis, we discovered that *CXCL13* is mainly produced by specific helper T cells and by exhausted immune cells. These results suggest that *CXCL13* could serve as a marker to identify patients whose tumors contain a more active immune environment. In the future, measuring *CXCL13* might help guide treatment decisions and improve personalized care for people with endometrial cancer. However, further studies using tissue samples and clinical trials are needed to confirm its usefulness.

## 1. Introduction

Endometrial carcinoma, also referred to as uterine corpus endometrial carcinoma (UCEC), is one of the most common malignancies of the female reproductive system and represents a growing global health burden. According to the Global Cancer Observatory of the International Agency for Research on Cancer, the estimated number of new corpus uteri cancer cases worldwide is projected to increase from 420,368 in 2022 to 676,296 in 2050, highlighting its increasing medical and social impact. In China, data from the National Cancer Registry reported approximately 77,000 new cases and 13,500 deaths in 2022, corresponding to incidence and mortality rates of 7.03 and 1.06 per 100,000 population, respectively [1]. Although patients diagnosed at an early stage generally achieve favorable outcomes after surgery and adjuvant therapy, advanced-stage UCEC remains associated with poor prognosis and limited therapeutic improvement [2,3,4]. The marked heterogeneity of molecular subtypes, treatment responses, and immune evasion patterns further underscores the need for reliable prognostic biomarkers and improved understanding of the tumor microenvironment (TME) in UCEC.

As research focus has shifted from tumor cells alone to the broader tumor microenvironment (TME), immune and stromal components have been increasingly recognized as key regulators of solid tumor progression, therapeutic response, and prognosis [5,6,7]. In UCEC, single-cell and immune-deconvolution studies have highlighted the importance of immune-cell exhaustion, cancer-associated fibroblast signaling, chemokine-mediated lymphocyte recruitment, and tertiary lymphoid structure (TLS)-associated immune activation in shaping antitumor immunity [8,9,10,11,12,13,14]. Among chemokines, *CXCL13* encodes C-X-C motif chemokine ligand 13, which binds CXCR5 and regulates the trafficking and spatial organization of B cells and follicular helper T cells, thereby contributing to lymphoid architecture and tumor immune responses [15]. However, the role of *CXCL13* appears to be highly context-dependent across tumor types: it has been associated with TLS formation and improved immunotherapy response in breast cancer, but also with regulatory B-cell recruitment and disease progression in prostate cancer [16,17]. In gynecologic tumors, single-cell profiling has identified CD8^+^ T cells as an important source of *CXCL13*, while UCEC-focused histological, genomic, and bioinformatic studies have reported associations among *CXCL13*, *CXCL9*, TLS-related immune features, favorable prognosis, and immune checkpoint gene expression [18,19,20,21]. Recent spatial transcriptomic work further supports the value of mapping *CXCL13*^+^ T cells and *CXCL9*^+^ antigen-presenting cells within tumor tissues, although the cellular sources and immune-regulatory relevance of *CXCL13* in the UCEC microenvironment remain incompletely defined [22].

Despite these observations, several questions remain unresolved. First, most available studies of *CXCL13* in UCEC have relied on bulk transcriptomic data, which cannot define the cellular source of *CXCL13* within the tumor immune microenvironment. Second, immune-cell populations such as CD8^+^ T cells, plasma cells, macrophage subsets, and TLS-associated signatures have often been discussed as biologically relevant features, but their relationships with *CXCL13* expression require more systematic evaluation using immune-deconvolution and single-cell approaches. Third, although *CXCL13* has been proposed as a prognostic biomarker, its association with immune scores, tumor-infiltrating immune-cell estimates, and pathway-level immune activation in UCEC has not been fully integrated in a single analytical framework [23,24,25].

Therefore, the present study aimed to evaluate the prognostic relevance of *CXCL13* and its association with immune microenvironmental features in UCEC using publicly available transcriptomic and single-cell datasets. We analyzed RNA-sequencing and clinical data from The Cancer Genome Atlas (TCGA) UCEC cohort, applied ESTIMATE and CIBERSORT to assess TME composition and tumor-infiltrating immune-cell estimates, performed survival and pathway enrichment analyses, and further used a public Gene Expression Omnibus (GEO) single-cell RNA-sequencing dataset to infer the cellular sources of *CXCL13*. This study was designed to clarify whether *CXCL13* is associated with prognosis and immune-active TME features in UCEC, while recognizing that transcriptomic findings require further histological, spatial, and functional validation [26,27,28].

## 2. Methods

### 2.1. Multi-Omics Data Processing and Analysis

#### 2.1.1. Data Sources and Preprocessing

Transcriptomic profiles and matched clinical annotations were retrieved from The Cancer Genome Atlas (TCGA) portal. All datasets analyzed in this study were obtained from publicly available repositories; no in-house patient specimens, histological sections, immunohistochemical data, or newly generated sequencing data were included. A total of 589 RNA-seq datasets in fragments per kilobase of transcript per million mapped reads (FPKM) format from UCEC patients were incorporated, consisting of 35 normal tissue samples, 539 tumor tissue specimens, and 15 samples of other categories. Clinical variables, including survival time, FIGO (International Federation of Gynecology and Obstetrics) stage, and histological grade, were collected. Expression values were normalized using log_2_(FPKM + 1) [25,29].

#### 2.1.2. Calculation of Tumor Microenvironment Scores

The quantitative assessment of tumor microenvironment characteristics was performed using the ESTIMATE algorithm as implemented in the estimate R package (v4.4.2), which calculates ImmuneScore, StromalScore, and the composite ESTIMATE score [25,30]. Each of these scores demonstrated a strong positive correlation with the corresponding cellular component proportions, with Pearson correlation coefficients exceeding 0.85 (*p* < 0.001) [30].

#### 2.1.3. Baseline Clinical Characteristics Analysis

To address baseline comparability across expression-defined groups, 539 TCGA-UCEC tumor samples were stratified into high- and low-*CXCL13* expression groups according to the median *CXCL13* expression value. Baseline clinicopathological characteristics, including age, FIGO stage, residual disease, tumor grade, histological subtype, survival status, overall survival time, and integrative molecular cluster, were summarized for the total cohort and for each *CXCL13* expression group. Continuous variables were presented as median with interquartile range and compared using the Wilcoxon rank-sum test. Categorical variables were presented as number and percentage and compared using the chi-square test. Missing or unknown values were excluded from statistical comparisons. A two-sided *p*-value < 0.05 was considered statistically significant.

#### 2.1.4. Survival Analysis

Survival differences between high-score and low-score groups were assessed using the Kaplan–Meier method, based on a cohort of 583 patients with complete follow-up records spanning 0 to 18.8 years. Statistical significance was evaluated using the log-rank test, with a threshold of *p* < 0.05. Hazard ratios (HR) and their 95% confidence intervals were derived from the Cox proportional hazards model [25,26].

#### 2.1.5. Identification of Differentially Expressed Genes Between High- and Low-Score Groups for ImmuneScores and StromalScores

Following stratification of samples by median scores for immune and stromal components, differential expression analysis was conducted using the limma package (v3.56.2), applying thresholds of ∣log_2_FC∣ > 1 and FDR < 0.05 [31]. This approach yielded 170 immune-related DEGs (differentially expressed gene) (143 up- and 27 down-regulated) and 1013 stromal-related DEGs (901 up- and 112 down-regulated).

#### 2.1.6. GO and KEGG Enrichment Analysis

Functional enrichment analysis was performed on the 147 overlapping DEGs using the clusterProfiler, enrichplot, and ggplot2 packages. The analysis covered Gene Ontology (GO) terms across biological processes, molecular functions, and cellular components, as well as KEGG (Kyoto Encyclopedia of Genes and Genomes) pathway annotations. A corrected *p*-value threshold of <0.05 was applied to determine statistical significance [32,33].

#### 2.1.7. Heatmap

Visualization of differentially expressed gene (DEG) expression patterns was achieved using the heatmap package within R. To explore variations in score distributions across different clinical stages, clinicopathological data for UCEC samples were retrieved from TCGA. Statistical comparisons between groups were performed in R using either the Wilcoxon test or the Kruskal–Wallis test, depending on the number of groups being compared.

#### 2.1.8. PPI Network Construction

A protein–protein interaction (PPI) network was constructed using the STRING database (v11.5), applying an interaction confidence threshold of >0.95. Visualization of the core network modules was carried out using Cytoscape (v3.10.3). Subsequent univariate Cox regression analysis (with criteria of *p* < 0.01, HR < 2) identified nine hub genes, including *CXCL13* [34,35].

#### 2.1.9. Cox Regression Analysis

Univariate Cox regression analysis was performed using the survival package in R. This analysis yielded 48 genes that satisfied the screening criteria of a *p*-value < 0.01 and an HR < 2.

#### 2.1.10. Gene Set Enrichment Analysis

Gene set enrichment analysis (GSEA) was conducted using RStudio (RStudio 2025 05.1 + 513), with reference to the Hallmark and C7 gene set collections (v7.0) from the Molecular Signatures Database. The analysis was applied to the full transcriptome profiles of all tumor samples, and significance was determined using a nominal *p*-value < 0.05 and a false discovery rate (FDR) q-value < 0.05.

#### 2.1.11. Tumor-Infiltrating Cell Abundance Profiles

The CIBERSORT algorithm was applied to estimate the relative fractions of tumor-infiltrating immune cell (TIC) populations from bulk RNA-seq profiles across tumor samples. Therefore, immune-cell abundances reported in this study should be interpreted as transcriptome-inferred estimates rather than histologically or immunohistochemically measured cell counts. Following quality control procedures, the final CIBERSORT-based analysis was conducted on 539 tumor specimens.

### 2.2. Processing and Analysis of Single-Cell RNA Sequencing Data

To infer the cellular sources of *CXCL13*, we retrieved and processed the publicly available single-cell RNA sequencing (scRNA-seq) dataset GSE173682 from the Gene Expression Omnibus (GEO) database [18]. This dataset includes endometrial cancer-associated samples as well as additional gynecological or related tumor samples, and provides single-cell transcriptomic profiles for downstream cell-type annotation and *CXCL13* expression analysis.

#### 2.2.1. Clinical Characteristics of the Single-Cell Dataset

The public scRNA-seq dataset GSE173682 incorporated in this study includes 11 samples, among which cases 1–5 correspond to primary endometrial cancer, case 6 represents endometrial cancer with ovarian metastasis, and cases 7–11 include other gynecological or related tumor samples that were used as background or comparative samples, consistent with the original dataset annotation [18]. To assess the applicability of the single-cell findings to the TCGA cohort, a detailed statistical evaluation of the clinical characteristics of these 11 patients was conducted (Table 1). Among them, 6 patients (54.5%) were classified as FIGO Stage I, 1 (9.1%) as Stage II, 2 (18.2%) as Stage III, and 2 (18.2%) as Stage IV. Compared with the TCGA reference cohort employed in this analysis (*n* = 539; stage I, 56.2%; stage II, 7.7%; stage III, 18.7%; stage IV, 3.7%), the FIGO stage distribution of the single-cell cohort showed no statistically significant difference (*p* = 0.129, chi-square test). Similarly, patient age did not differ significantly (*p* = 0.586, Wilcoxon test). These findings indicate that the selected single-cell cohort is highly consistent with the larger TCGA cohort in terms of clinical and pathological features, is representative of the broader UCEC patient population, and is suitable for subsequent validation of cellular origins and microenvironmental regulatory mechanisms.

The table summarizes the clinical information and single-cell sequencing data for the 11 samples from the GSE173682 dataset. Cases 1–5 correspond to primary endometrial cancer; case 6 represents endometrial cancer with ovarian metastasis; and cases 7–11 consist of samples from other gynecological tumor types, which were included as background or control groups in this study. Tumor staging was determined according to the FIGO classification system (8th edition). Age is expressed in years, while body mass index (BMI) is presented in kg/m^2^. Within the scATAC-seq and scRNA-seq columns, values outside the square brackets indicate the number of cells retained following initial quality control procedures, whereas values inside the brackets denote the number of cells derived from the raw sequencing data. Following stringent quality filtering (nFeature_RNA ranging from 300 to 10,000, nCount_RNA exceeding 600, mitochondrial gene content below 10%, and hemoglobin gene content below 1%), along with the removal of duplicate cells, a final set of 34,312 high-quality cells was obtained for subsequent clustering analysis. The ordering of data within this table follows the methodology outlined by Regner et al. (2021) [18].

#### 2.2.2. Data Quality Control and Standardization

Initial quality control was carried out using the Seurat package (v4.3) [28], with cells removed based on the following criteria: nFeature_RNA < 300 or >10,000; nCount_RNA < 600; and mitochondrial gene content exceeding 10%. This process yielded 34,312 high-quality cells for subsequent analyses. Normalization was performed via the SCTransform method, which mitigates technical variation while stabilizing variance [8,28].

#### 2.2.3. Batch Effect Correction and Dimension Reduction/Clustering

To evaluate potential batch effects among samples, dimensionality reduction was first performed using principal component analysis (PCA), and Harmony correction was additionally tested as a sensitivity assessment. Cells from different samples showed a naturally mixed distribution in the original PCA space, indicating no obvious sample-driven batch clustering. In contrast, Harmony correction produced apparent over-correction in some samples, characterized by artificial separation of cell populations. Therefore, PCA-based embeddings were used for the primary downstream dimensionality reduction and clustering analyses, while Harmony-corrected results were not used as the main analytical basis.

The specific workflow is as follows: Principal Component Analysis (PCA) was performed, and the top 40 principal components were selected. A k-nearest neighbors (k-NN) plot was constructed based on the PCA results, and cell clustering was performed using the Louvain algorithm (with a resolution set to 1.0). Finally, the cell population structure was visualized using the UMAP (Unified Manifold Approximation and Projection) nonlinear dimension reduction method [8], and a t-SNE dimension reduction plot was generated simultaneously for supplementary validation. By comparing clustering results at different resolutions (0.1–2.0), a resolution of 1.0 was determined to be the optimal parameter, balancing the precision of cell subpopulation identification with biological interpretability.

#### 2.2.4. Cell Type Annotation

A two-pronged strategy was adopted for cell type identification: (1) automated annotation using the SingleR software (v2.4.1) against the Human Primary Cell Atlas [28]; and (2) manual annotation based on established lineage-specific marker genes, including those for T cells, macrophages, endothelial cells, tissue stem cells, epithelial cells, smooth muscle cells, mesenchymal stem cells (MSCs), fibroblasts, and natural killer (NK) cells [8,18]. Ultimately, cells were classified into nine major categories: T cells, macrophages, endothelial cells, tissue stem cells, epithelial cells, smooth muscle cells, MSCs, fibroblasts, and NK cells (natural killer cell).

#### 2.2.5. Detailed Analysis of T Cell Subpopulations

To explore the heterogeneity of T cells within the tumor microenvironment, a subset of 9842 T cells was re-clustered (resolution = 0.6), which yielded 16 distinct functional subpopulations. T cell subtypes were assigned according to characteristic gene expression signatures: follicular helper T cells (Tfh) defined by *CXCL13*, *BCL6*, and *CD27*; exhausted CD8^+^ T cells marked by *PDCD1*, *LAG3*, and *TIGIT*; regulatory T cells (Treg) characterized by *FOXP3*, *IL2RA*, and *CTLA4*; and tissue-resident memory T cells (Trm) identified by *ITGAE*, among others [8,10].

#### 2.2.6. Analysis of CXCL13 Expression

The expression profiles and spatial distribution of *CXCL13* across different cell types and T-cell subsets were visualized using FeaturePlot, VlnPlot, and DotPlot. Spearman’s rank correlation coefficient was used to evaluate the relationship between *CXCL13* transcript levels and the relative proportions of individual cell types. Comparisons of *CXCL13* expression between distinct cell populations were performed using the Wilcoxon signed-rank test, with statistical significance set at *p* < 0.05 [8,18].

#### 2.2.7. Quality Control Standards

Strict quality control parameters were established for all analytical steps to ensure the reliability of the results. Cell doublets were identified and removed using DoubletFinder software (v2.0.3), and the effects of the cell cycle were corrected via regression analysis [8,28].

## 3. Results

### 3.1. Analytical Workflow of This Study

This study primarily consists of two analytical workflows. The first involves analyzing TCGA multi-omics data to identify and validate core prognostic genes for UCEC; the second involves processing and analyzing single-cell RNA sequencing data.

#### 3.1.1. Identification and Validation of Core Prognostic Genes for Endometrial Cancer (UCEC) Based on TCGA Multi-Omics Data

**Figure 1:**
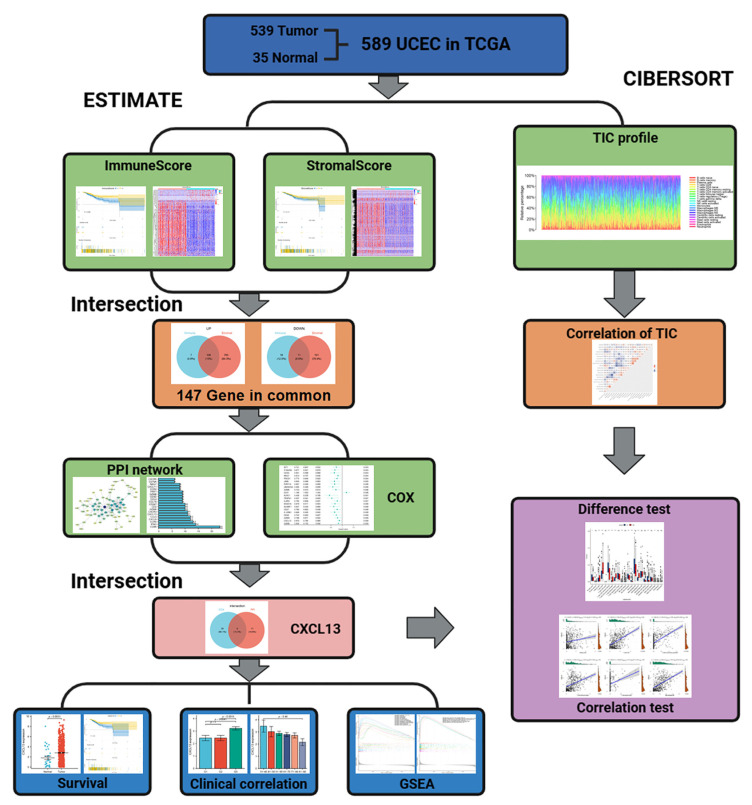
Analysis of TCGA Multi-omics Data to Identify and Validate Prognostic Hub Genes in UCEC.

The analytical pipeline used to screen and validate hub prognostic genes in UCEC using TCGA multi-omics datasets is illustrated in Figure 1. RNA-sequencing profiles of 589 UCEC samples were downloaded from TCGA, and the CIBERSORT and ESTIMATE algorithms were applied to quantify the fractions of tumor-infiltrating immune cells (TICs) as well as the levels of immune and stromal components. After identifying differentially expressed genes (DEGs) common to both ImmuneScore and StromalScore, a protein–protein interaction (PPI) network was constructed, followed by univariate Cox regression analysis to pinpoint prognosis-associated core genes. Intersection analysis yielded nine core genes, with *CXCL13* among them. This gene was subsequently subjected to a multi-level validation process, including survival evaluation (Kaplan–Meier and Cox regression), assessment of clinicopathological correlations (Wilcoxon or Kruskal–Wallis tests), gene set enrichment analysis (GSEA), and immune infiltration correlation analysis using CIBERSORT. Through this systematic approach, we aimed to elucidate the potential of *CXCL13* as a prognostic biomarker and its regulatory roles within the tumor microenvironment (TME).

#### 3.1.2. Processing and Analysis of Single-Cell RNA Sequencing Data

**Figure 2:**
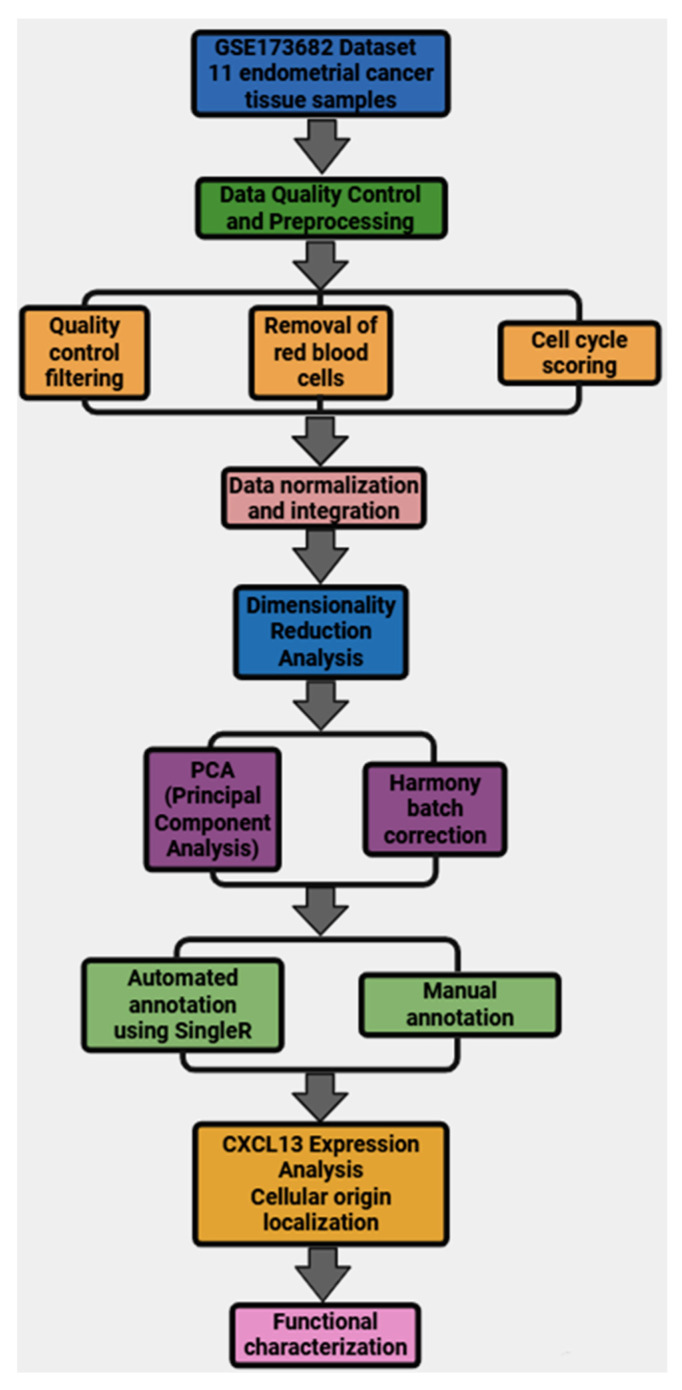
Schematic overview of the single-cell RNA-sequencing data processing and analysis pipeline.

Figure 2 outlines the workflow used to process and analyze the public scRNA-seq dataset GSE173682. This dataset includes endometrial cancer-associated samples as well as additional gynecological or related tumor samples, collectively providing 92,437 single-cell transcriptomic records in the original dataset. After quality control, normalization, and doublet removal, 34,312 high-quality cells were retained for downstream analysis. Principal component analysis (PCA) was used for the primary dimensionality reduction and clustering workflow, as batch-effect assessment did not indicate obvious sample-driven clustering in the original PCA space. Cells were then visualized using UMAP and annotated into nine major cell types based on automated annotation and canonical marker genes.

To infer the potential cellular sources of *CXCL13*, T-cell subsets were further re-clustered and annotated according to established marker-gene signatures. The distribution of *CXCL13* expression across major cell types and T-cell subsets was evaluated using FeaturePlot, VlnPlot, DotPlot, and correlation analysis. These analyses were used to characterize cell-type-associated *CXCL13* expression patterns rather than to provide histological validation of immune-cell infiltration.

The diagram outlines the sequential steps used to process and interpret the scRNA-seq dataset.

### 3.2. Baseline Clinicopathological Characteristics of the TCGA UCEC Cohort

A total of 539 TCGA-UCEC tumor samples were stratified into high- and low-*CXCL13* expression groups according to the median *CXCL13* expression value. The high-*CXCL13* group included 270 patients, while the low-*CXCL13* group included 269 patients. Baseline clinicopathological characteristics are summarized in Table 2.

Age, FIGO stage, residual disease status, histological subtype, vital status, and overall survival time were generally comparable between the two groups. However, tumor grade differed significantly between groups, with the high-*CXCL13* group showing a higher proportion of G3 tumors. The distribution of integrative molecular clusters also differed significantly between the high- and low-*CXCL13* groups. These findings indicate that clinicopathological and molecular background factors, particularly tumor grade and molecular subtype, should be considered when interpreting the prognostic and immune-microenvironment associations of *CXCL13*.

### 3.3. Association Between Tumor Microenvironment Scores and Clinicopathological Features in UCEC

**Figure 3:**
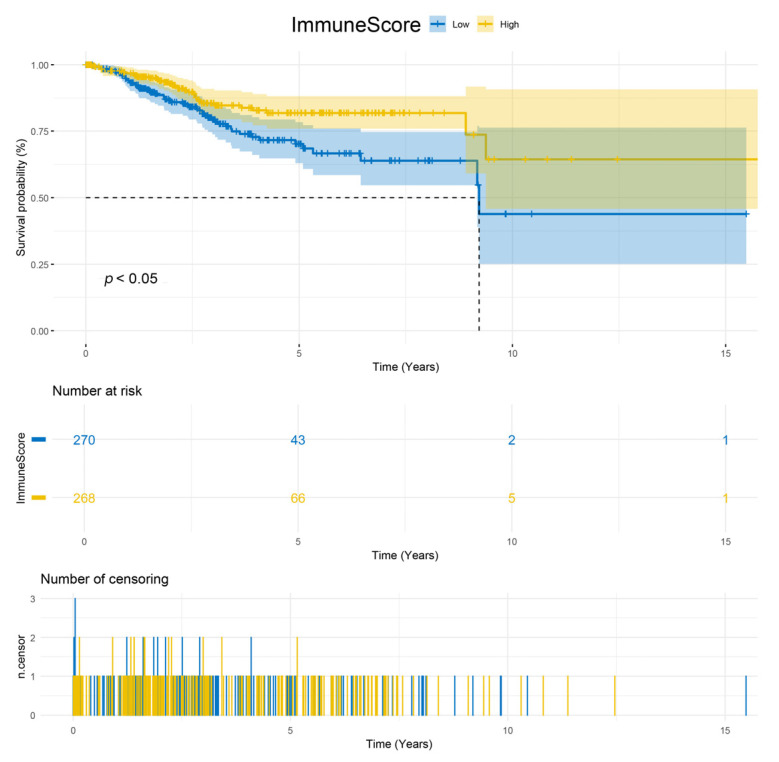
Association between ImmuneScore and overall survival in UCEC patients.

**Figure 4:**
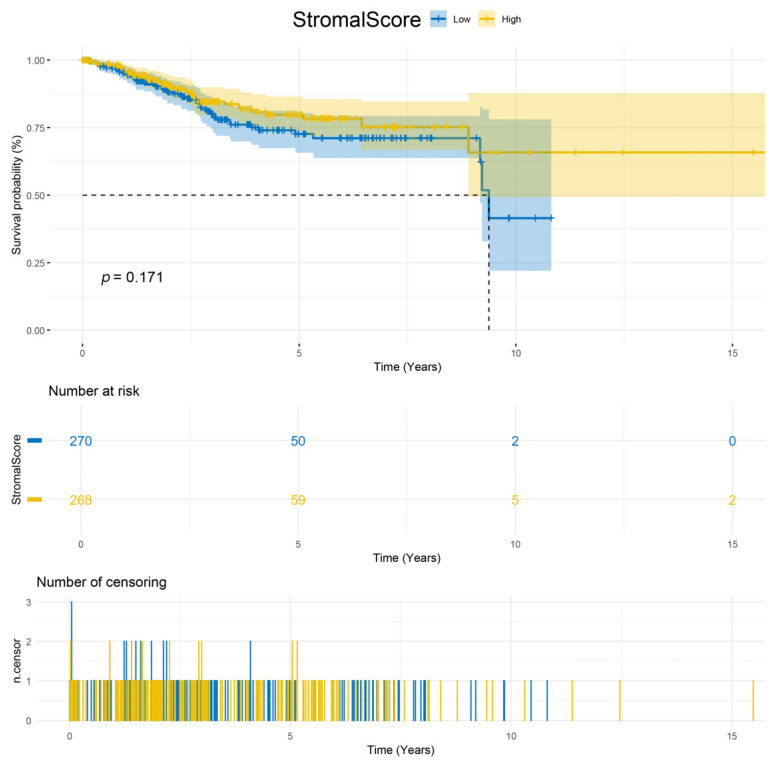
Association between StromalScore and overall survival in UCEC patients.

**Figure 5:**
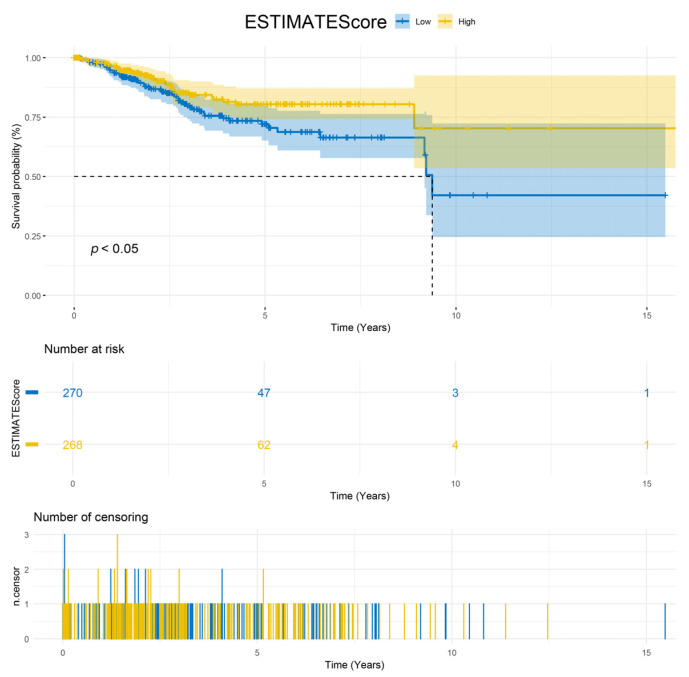
Association between ESTIMATEScore and overall survival in UCEC patients.

Our survival analysis demonstrated that a higher abundance of immune components within the tumor microenvironment (TME) correlates with a significantly better prognosis for UCEC patients. Using Kaplan-Meier curves and log-rank tests, we evaluated overall survival (OS) according to ImmuneScore, StromalScore, and the combined ESTIMATE score. Patients in the high-ImmuneScore stratum experienced markedly prolonged OS compared with their low-score counterparts (*p* < 0.05, Figure 3). In contrast, the StromalScore showed no significant relationship with OS (*p* = 0.171, Figure 4). Interestingly, the ESTIMATE score (calculated as ImmuneScore + StromalScore) remained an independent predictor of favorable outcome for the high-scoring group (*p* < 0.05, Figure 5). These results indicate that immune-related, rather than stromal, components are the primary determinants of prognosis within the TME. Consequently, the ImmuneScore may serve as a more reliable metric for predicting survival outcomes in endometrial cancer patients.

Patients were divided into low- and high-ImmuneScore groups based on the median value. The solid line shows the estimated survival probability, and the dashed lines represent the 95% confidence intervals (lower and upper limits). Kaplan–Meier curves with log-rank testing demonstrated a significantly longer overall survival in the high-score cohort (*p* < 0.05).

Patients were divided into low- and high-StromalScore groups based on the median value. Log-rank analysis of Kaplan–Meier survival estimates showed no statistically significant difference between the two groups (*p* = 0.17).

Patients were divided into low- and high-ESTIMATEScore groups based on the median value. Kaplan–Meier analysis followed by the log-rank test revealed a significant survival advantage for patients in the high-score group (*p* < 0.05).

### 3.4. The Ratio of Immune to Stromal Components Is Associated with the Clinical-Pathological Staging of Endometrial Cancer Patients

**Figure 6:**
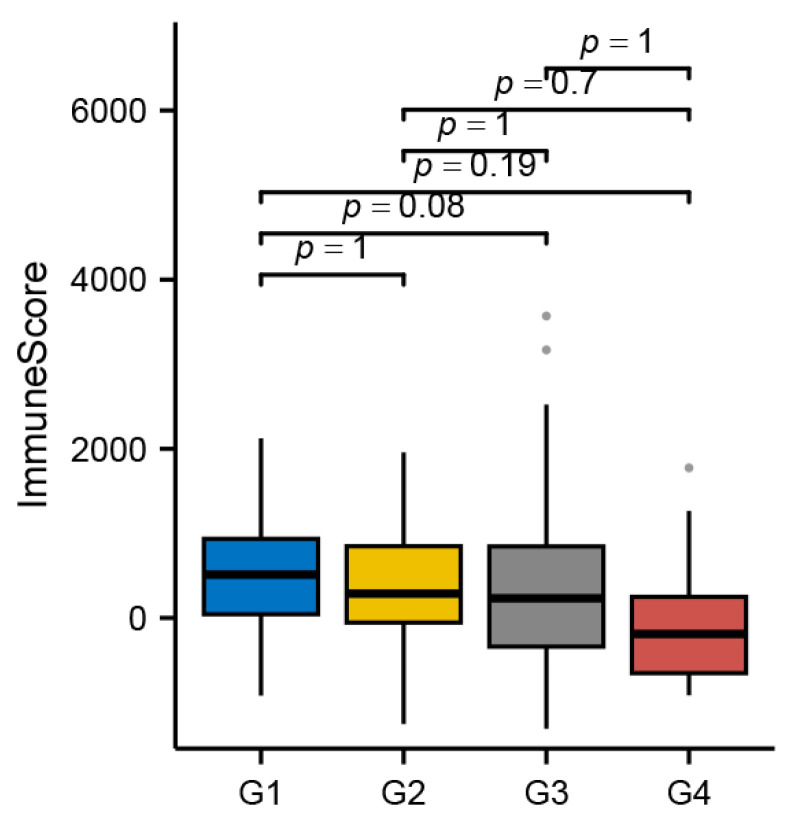
Correlation between immune scores and histological grading (Grade G).

**Figure 7:**
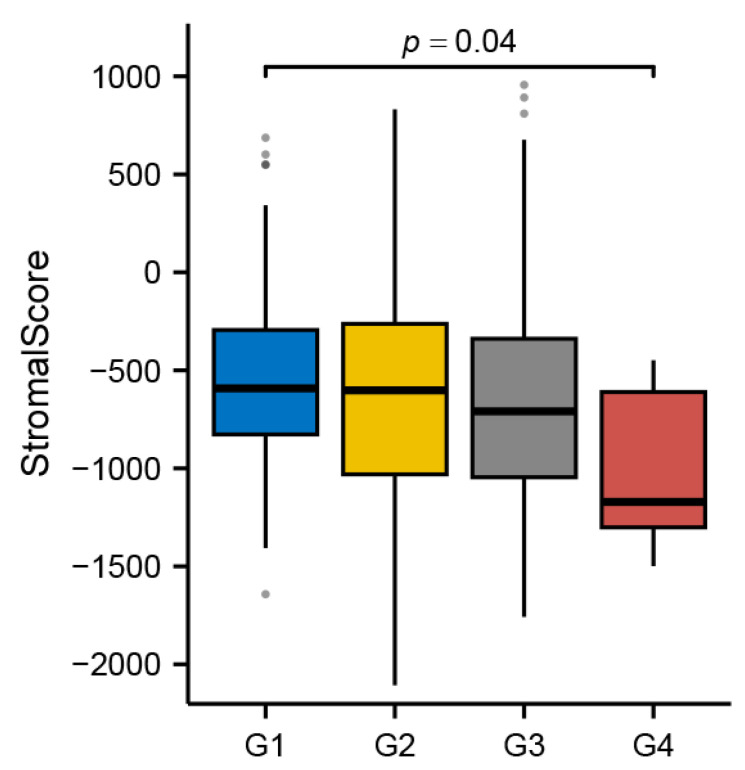
Correlation between StromalScore and histological grade (G grade).

**Figure 8:**
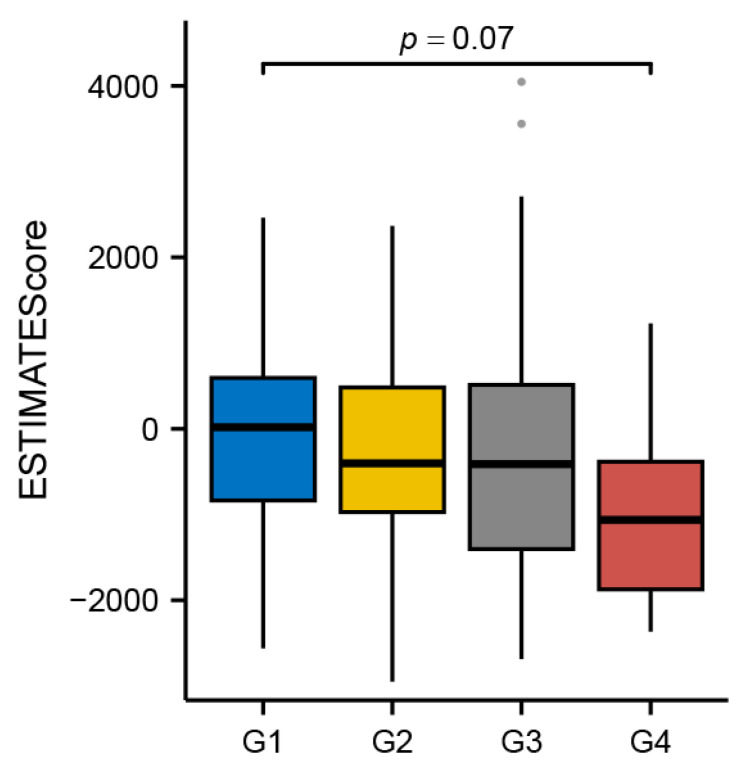
Correlation between the ESTIMATE score and histological grade (G grade).

Analysis of clinical staging correlations indicates that a reduction in the immune-to-stromal ratio within the tumor microenvironment (TME) is closely associated with the malignant progression of endometrial cancer (UCEC). Based on TCGA clinical-pathological data, the ImmuneScore showed a weak negative correlation with histological grade (G grade) (*p* = 0.19, Figure 6), while the StromalScore decreased as G grade increased (*p* = 0.04, Figure 7). The ESTIMATE composite score (ImmuneScore + StromalScore) was negatively correlated with histological grade (G grade), and this score was significantly lower in metastatic tumors (*p* = 0.07, Figure 8). This downward gradient suggests that the depletion of immune/stromal components in the TME may drive the evolution of an aggressive tumor phenotype.

According to the Kruskal–Wallis rank-sum test, the *p*-value for the immune score was 0.19. Each grey dot represents the ImmuneScore of an individual tumor sample (*n* = 539) from the TCGA-UCEC cohort.

The *p*-value for the StromalScore was 0.04, as determined by the Kruskal–Wallis rank-sum test. Each grey dot represents an individual tumor sample (*n* = 539).

The *p*-value for the ESTIMATE score was 0.07, as determined by the Kruskal–Wallis rank-sum test. Each grey dot represents an individual tumor sample (*n* = 539).

### 3.5. Analysis of Differentially Expressed Genes Common to Both ImmuneScore and StromalScore Revealed a Marked Enrichment in Pathways Related to Immune Activation

**Figure 9:**
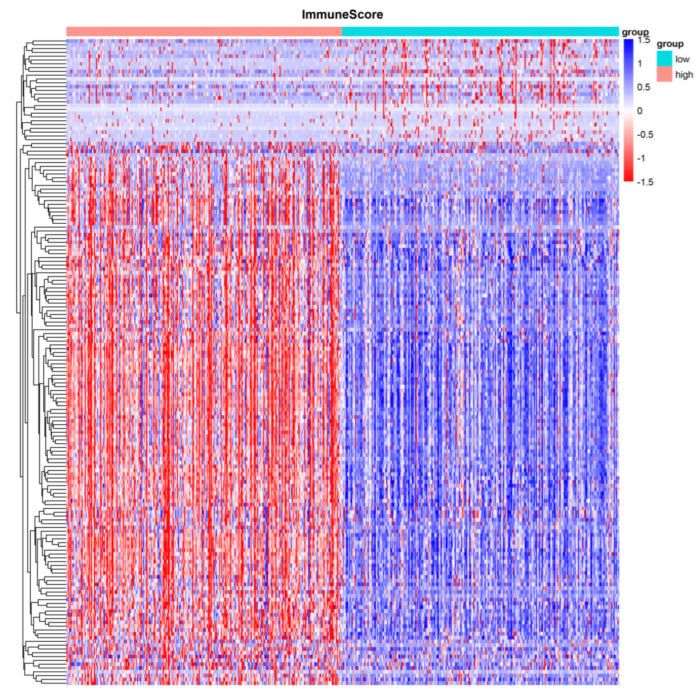
Heatmap visualization of differentially expressed genes (DEGs) identified by ImmuneScore.

**Figure 10:**
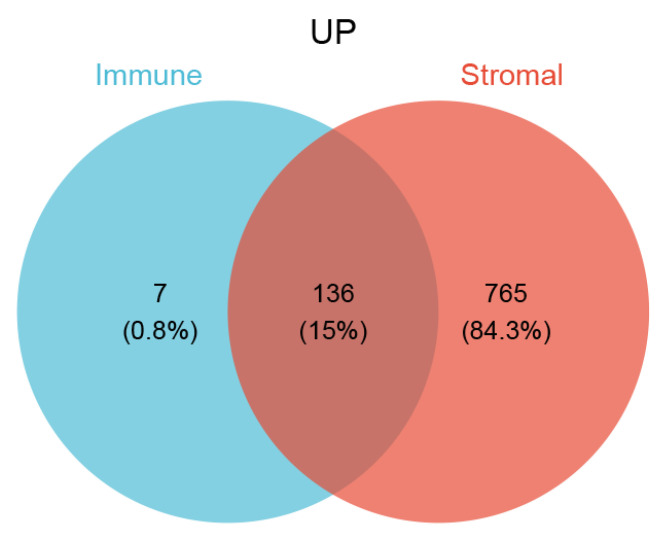
Venn diagram illustrating the overlap of upregulated DEGs shared between ImmuneScore and StromalScore.

**Figure 11:**
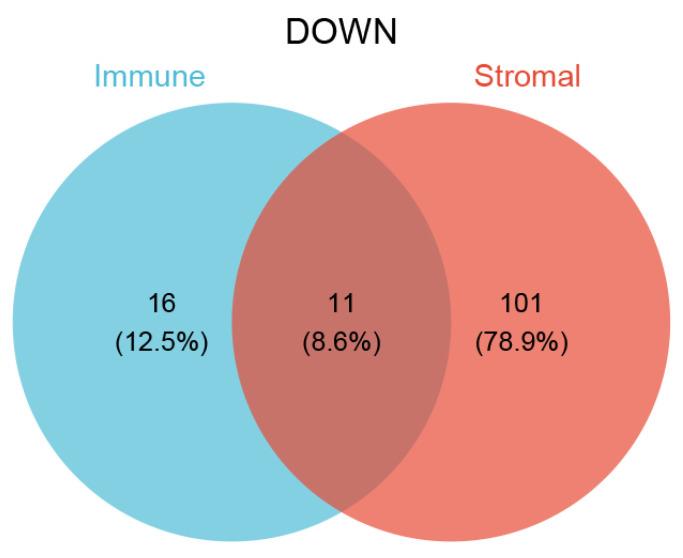
Venn diagram illustrating the overlap of downregulated DEGs shared between ImmuneScore and StromalScore.

**Figure 12:**
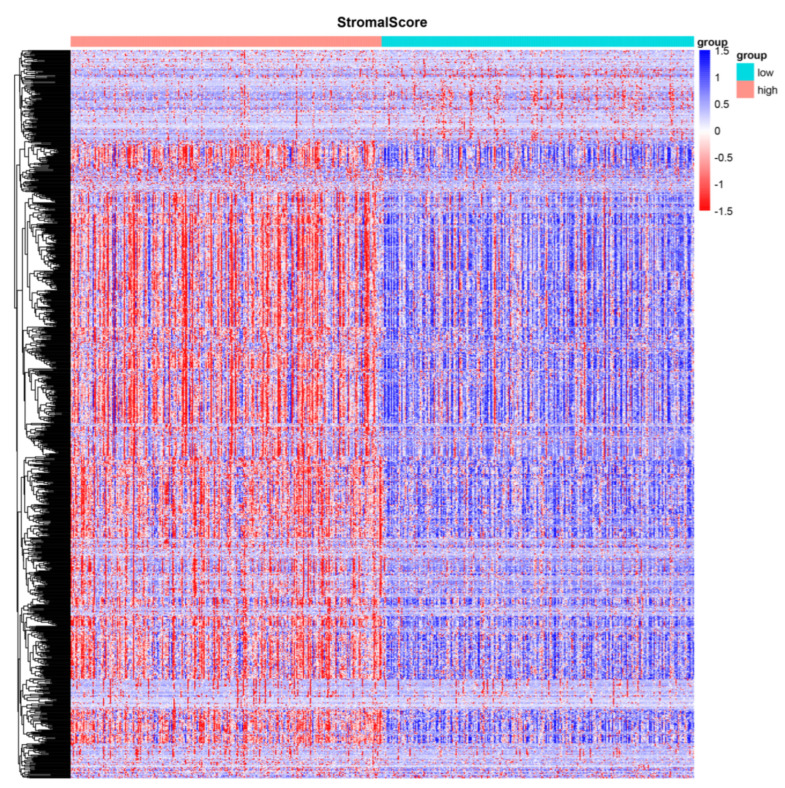
Heatmap visualization of differentially expressed genes (DEGs) identified by StromalScore.

**Figure 13:**
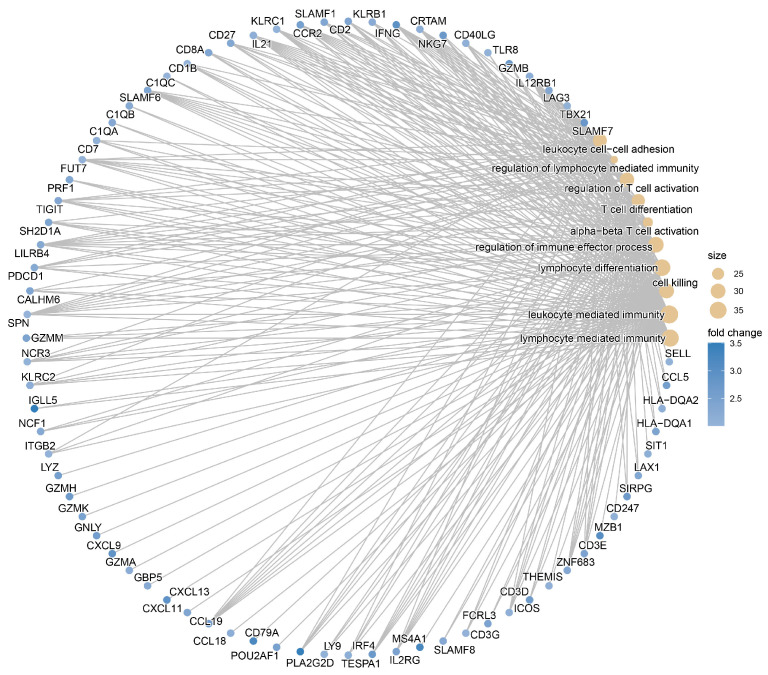
Gene Ontology (GO) enrichment analysis of the 147 DEGs common to both ImmuneScore and StromalScore.

**Figure 14:**
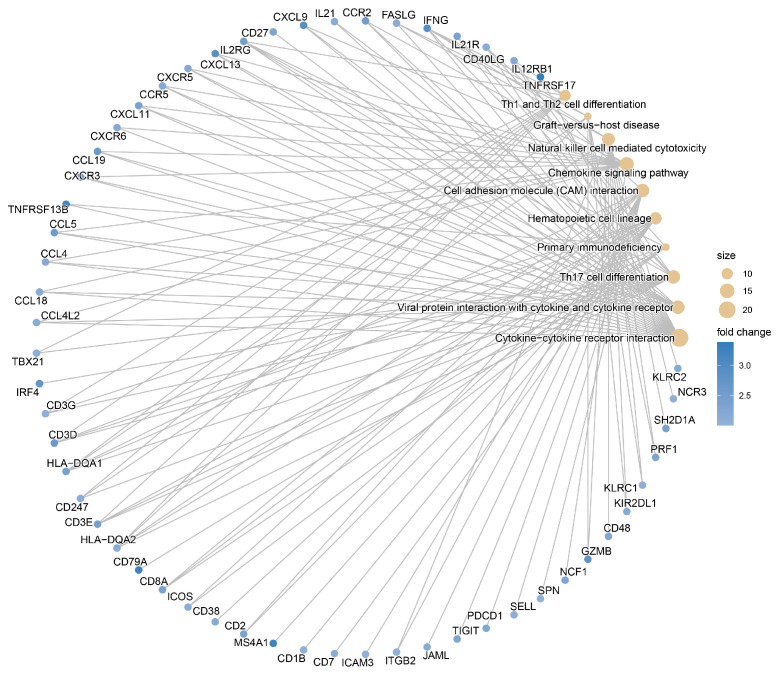
Kyoto Encyclopedia of Genes and Genomes (KEGG) pathway enrichment analysis of the 147 DEGs common to both ImmuneScore and StromalScore.

Comparison between the high- and low-ImmuneScore groups yielded 170 differentially expressed genes (DEGs). Among these, 143 upregulated genes—including *CXCL13* and *GZMB*—were mainly associated with immune activation pathways, whereas 27 downregulated genes (*p* = 1.2 × 10^−8^) were linked to immune suppression (Figure 9, Figure 10 and Figure 11). These observations suggest that the gene regulatory networks involving immune and stromal components within the tumor microenvironment (TME) are strongly oriented toward immune-related pathways. A more pronounced outcome was obtained from the StromalScore differential analysis, which identified 1013 DEGs (901 up, 112 down). The upregulated genes were predominantly involved in extracellular matrix remodeling (e.g., COL1A1, MMP9) (Figure 10, Figure 11 and Figure 12). Venn diagram cross-analysis showed that the high-score groups of both ImmuneScore and StromalScore shared 136 upregulated genes (representing 95.1% of the immune-associated upregulated set), while 11 downregulated genes overlapped in the low-score groups, indicating that immune components exert a dominant regulatory influence on TME-related genes (Figure 11 and Figure 12). Gene Ontology (GO) enrichment analysis of the 147 common DEGs revealed significant overrepresentation of immune-related biological processes, including leukocyte activation involved in immune response (FDR = 8.1 × 10^−9^) and T cell differentiation involved in immune response (FDR = 7.4 × 10^−7^) (Figure 13). Furthermore, KEGG pathway analysis highlighted the chemokine signaling pathway (hsa04062, FDR = 8.9 × 10^−8^) and cytokine-cytokine receptor interaction (hsa04060, FDR = 5.8 × 10^−13^) as key regulatory routes (Figure 14). Taken together, these results demonstrate that the TME characteristics of UCEC are predominantly shaped by immune-related genes rather than by stromal/matrix components.

Expression profiles were compared between the high-score and low-score groups. Rows represent individual genes, while columns correspond to sample identifiers (not displayed). DEGs were determined using the Wilcoxon rank-sum test, applying a significance cutoff of q = 0.05 and a log2-transformed fold change threshold > 1.

A total of 136 common upregulated genes were identified, accounting for 15% of the combined gene set across both scoring systems. The threshold for differential expression was established at q = 1.

Eleven overlapping downregulated genes were found, representing 8.6% of all DEGs from the two comparisons. Differential expression was defined using a threshold of q < 1.

Expression profiles were compared between the high-score and low-score groups. Rows represent individual genes, while columns correspond to sample identifiers (not displayed). DEGs were determined using the Wilcoxon rank-sum test, applying a significance cutoff of q = 0.05 and a log2-transformed fold change threshold > 1.

The analysis covered three main categories: biological process, molecular function, and cellular component. All displayed terms met the significance criteria (FDR q < 0.05 and *p* < 0.05).

All enriched pathways shown achieved statistical significance (FDR q < 0.05 and *p* < 0.05).

### 3.6. Integrative Analysis of Protein–Protein Interaction Network and Univariate Cox Regression

**Figure 15:**
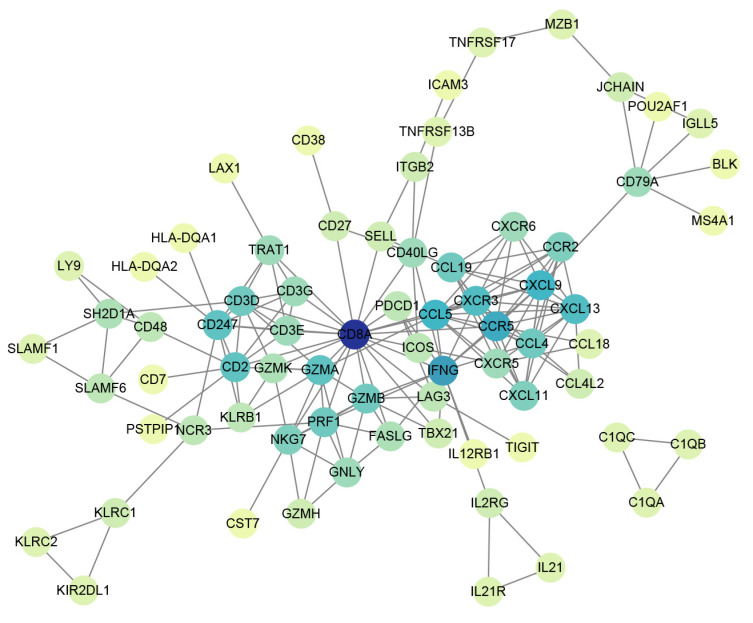
Protein–protein interaction network.

**Figure 16:**
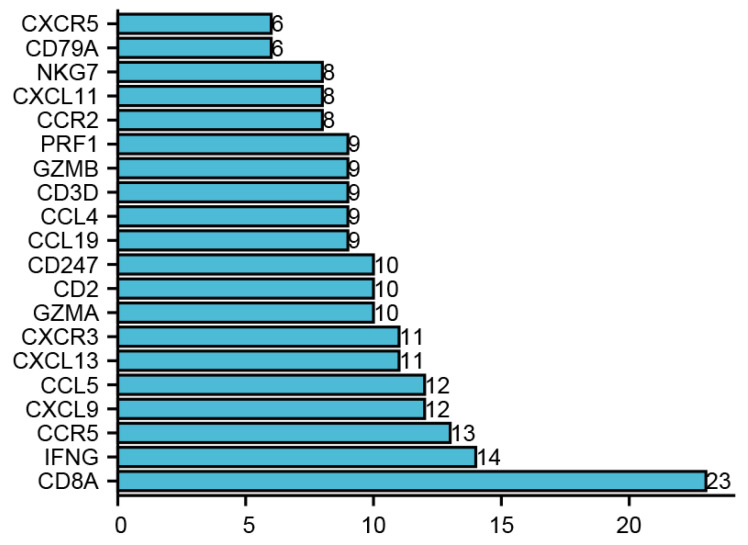
Bar chart showing the top 20 genes in the protein–protein interaction (PPI) network, sorted by degree.

To further explore the molecular regulatory mechanisms driving endometrial cancer (UCEC), a protein–protein interaction (PPI) network was constructed using the STRING database (v11.5) with a confidence threshold above 0.95. The resulting network was then visualized via Cytoscape (v3.10.3). As presented in Figure 15, this network consisted of 72 gene nodes and their corresponding interactions, while the connectivity distribution of the top 20 genes, ranked by degree, is summarized in the histogram shown in Figure 16.

**Figure 17:**
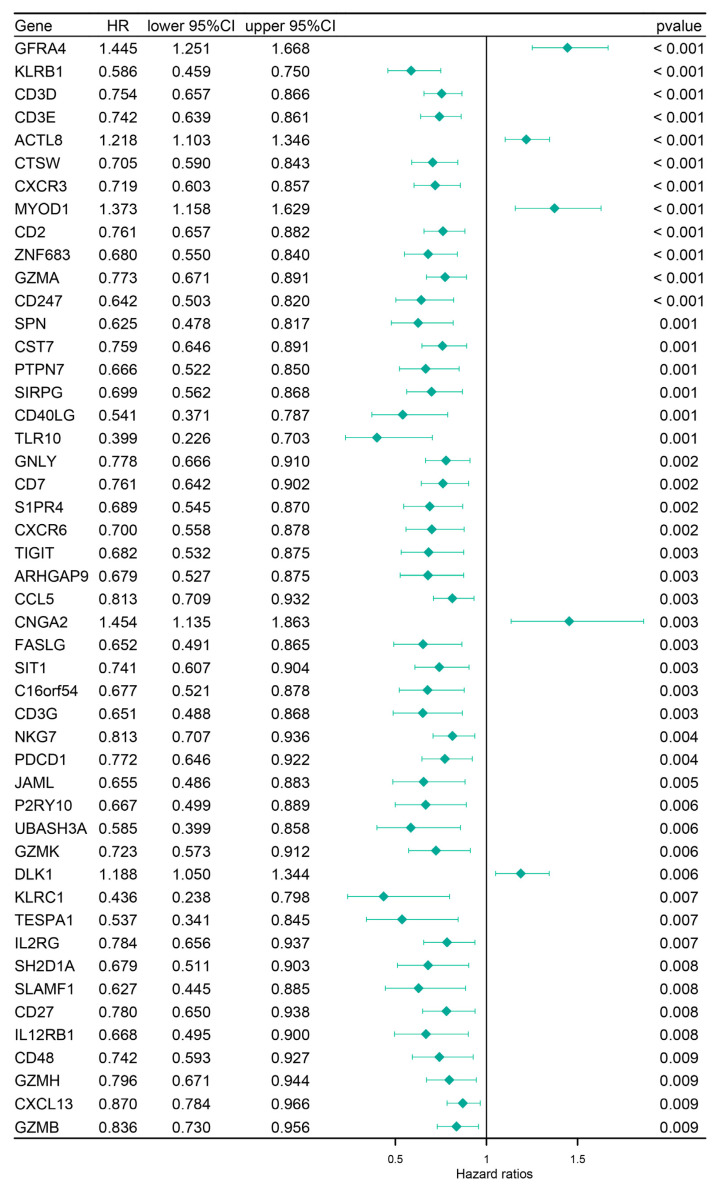
Univariate Cox regression analysis of differentially expressed genes (DEGs).

**Figure 18:**
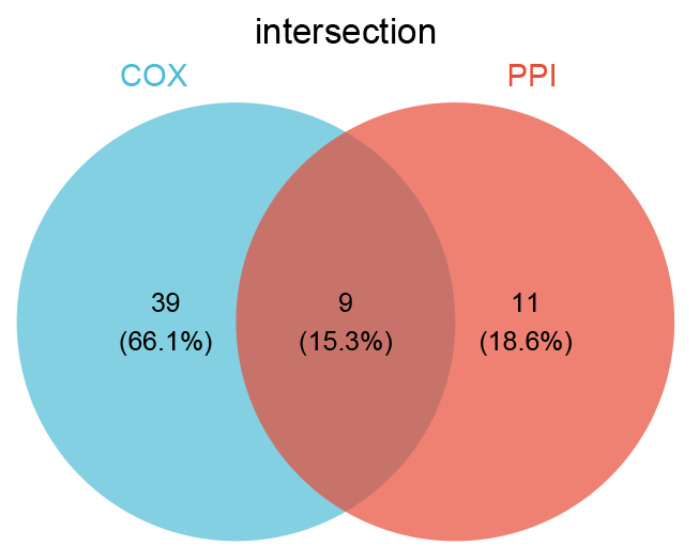
Venn diagram illustrating the intersection between protein–protein interaction (PPI) network genes and those identified by univariate Cox regression analysis.

Subsequently, univariate Cox regression analysis was performed on the 147 differentially expressed genes (DEGs) using significance thresholds of *p* < 0.01 and a hazard ratio (HR) below 2. This approach identified 48 genes significantly associated with patient survival (Figure 17). To cross-validate these results, the core node genes from the PPI network were intersected with the Cox regression findings. Through this integrative strategy, nine key hub genes were ultimately identified: *CXCL13*, *GZMB*, *NKG7*, *CCL5*, *CD247*, *GZMA*, *CD2*, *CXCR3*, and *CD3D* (Figure 18). These genes play critical roles in immune activation and T-cell signaling pathways, suggesting that they may influence patient outcomes by reshaping the immunophenotype of the tumor microenvironment (TME).

This interaction network is constructed from nodes with interaction confidence scores greater than 0.95.

The top 20 genes in the protein–protein interaction network are sorted by degree.

Univariate Cox regression was conducted on 147 differentially expressed genes, among which 48 met a significance threshold of *p* < 0.01 and were found to be significantly associated with patient survival.

A total of nine genes were common to the top 20 genes of the PPI network and the 48 genes found to be significantly associated with patient survival via univariate Cox regression, representing 15.3% of all differentially expressed genes.

### 3.7. Association of CXCL13 Expression with TLS-Related Immune-Activation Features

To further interpret the immune-active phenotype associated with *CXCL13* expression, we examined whether the *CXCL13*-high transcriptional profile was consistent with previously reported tertiary lymphoid structure (TLS)-related immune features. In the present TCGA-based analysis, high *CXCL13* expression was accompanied by enrichment of immune-activation pathways and increased transcriptome-inferred immune-cell fractions, including CD8^+^ T cells and plasma cells. Several TLS-associated chemokine and lymphocyte-organization genes, including *CXCL13*, *CXCL9*, *LTB*, and *LTA*, also showed higher expression in the immune-active subgroup, suggesting that *CXCL13*-high tumors may exhibit transcriptional features compatible with TLS-associated antitumor immunity.

This interpretation is supported by previous tissue-based and spatial studies. Nagase et al. reported that highly immunogenic endometrial carcinoma samples showed increased TLS abundance together with coordinated elevation of *CXCL13* and *CXCL9*, and that *CXCL13*-positive follicular dendritic cells were mainly localized within B-cell zones of TLS regions [20]. In parallel, Wang et al. reported that elevated *CXCL13* mRNA expression was associated with favorable prognosis and immune checkpoint gene expression in UCEC based on TCGA and GEO datasets [21]. Spatial transcriptomic work by Kobayashi et al. further emphasized the importance of mapping *CXCL13*^+^ T cells and *CXCL9*^+^ antigen-presenting cells within tumor tissues during antitumor immune responses [22].

However, because the present study did not include hematoxylin-eosin staining, immunohistochemistry, multiplex immunofluorescence, or spatial transcriptomic validation, TLSs were not directly identified in our cohort. Therefore, the current findings should be interpreted as transcriptomic evidence linking high *CXCL13* expression to TLS-related immune-activation features, rather than direct evidence that *CXCL13* induces TLS formation in UCEC.

### 3.8. Association Between CXCL13 Expression and Patient Survival as Well as Pathological Grade in Endometrial Cancer

*CXCL13*, also referred to as B-cell chemoattractant (BCA-1 or BLC), orchestrates the directed migration and homing of B lymphocytes and follicular helper T (Tfh) cells through specific engagement with its receptor, *CXCR5*. Under physiological conditions, its expression is primarily derived from stromal cells within secondary lymphoid organs, including the spleen and lymph nodes. Within the tumor microenvironment (TME), *CXCL13* contributes to lymphocyte recruitment and the generation of tertiary lymphoid structures (TLSs), thereby modulating antitumor immune responses.

**Figure 19:**
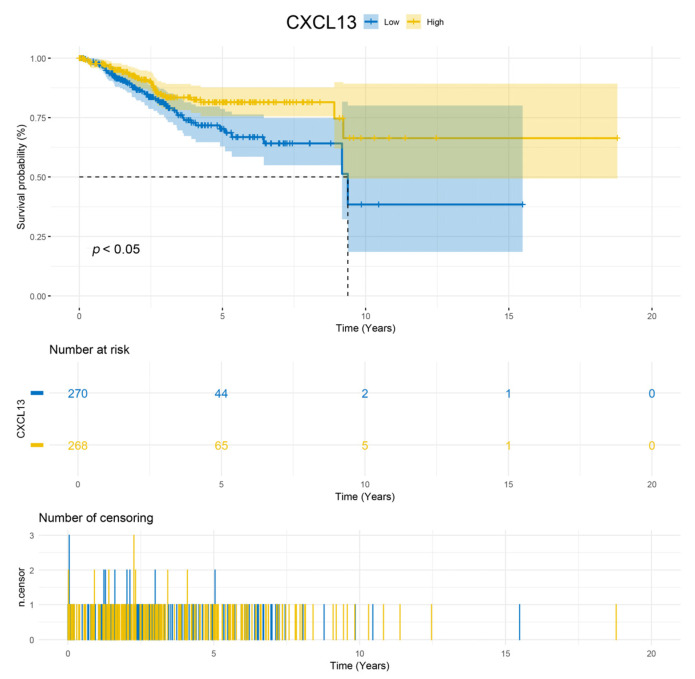
Relationship between *CXCL13* transcript levels and overall survival in UCEC patients.

**Figure 20:**
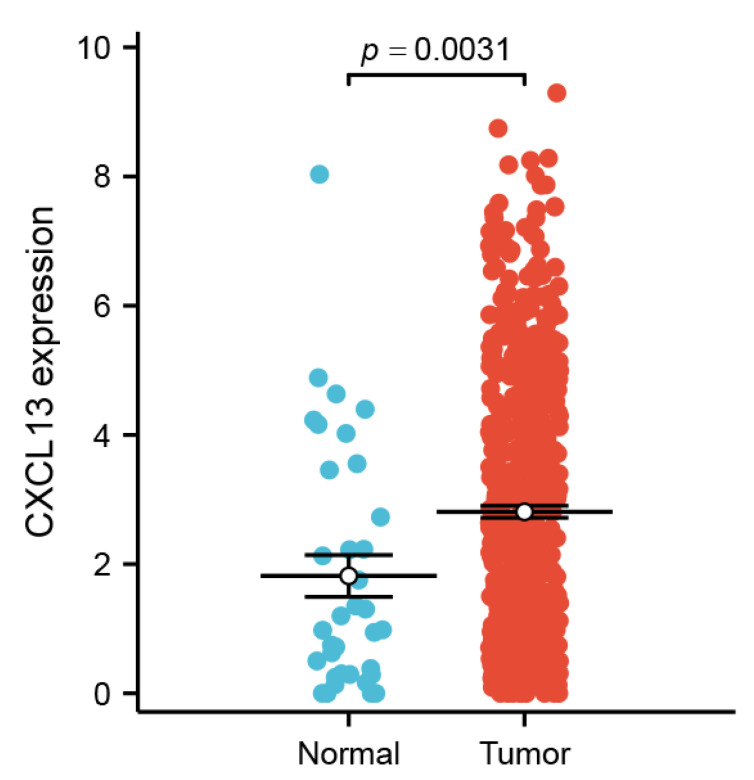
Comparison of *CXCL13* expression between normal and malignant endometrial tissues.

**Figure 21:**
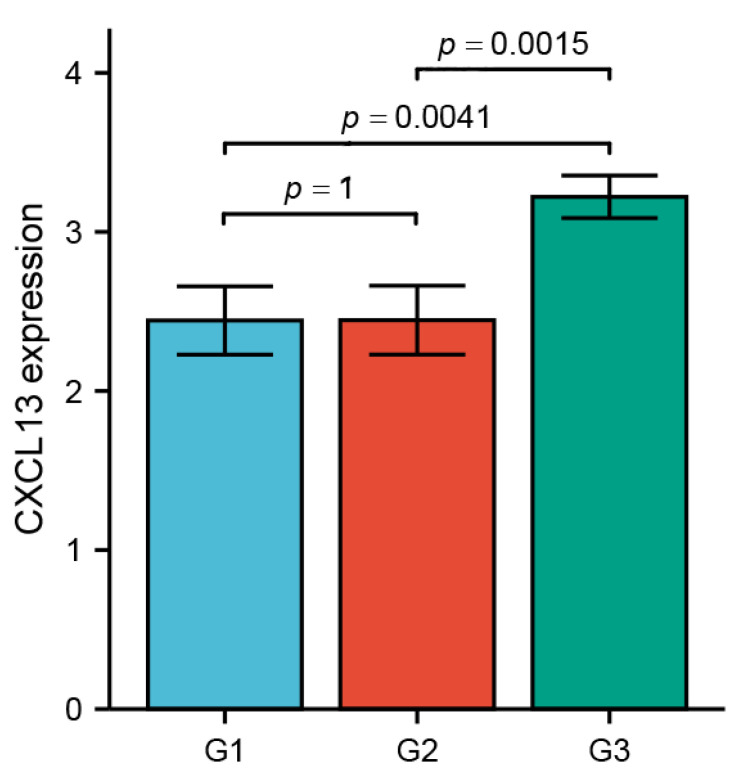
Association of *CXCL13* expression with histological grade in endometrial cancer (UCEC).

In this study, 589 endometrial cancer (UCEC) samples were dichotomized into high- and low-expression groups according to the median *CXCL13* level. Survival analysis using the Kaplan–Meier method revealed that patients in the high-expression cohort experienced significantly prolonged overall survival compared with those in the low-expression cohort (*p* < 0.05, Figure 19). The Wilcoxon rank-sum test indicated that *CXCL13* expression was markedly higher in tumor tissues relative to normal counterparts (*p* = 0.0031, Figure 20 and Tables S1–S3), suggesting a strong association with UCEC malignancy. Further examination of histological grade (G grade) showed no notable difference in *CXCL13* expression between G1 and G2 tumors (*p* = 1), whereas expression levels were significantly elevated in G3 compared with G1/G2 (*p* = 0.0041, Figure 21). This pattern implies that increased *CXCL13* expression may be linked to immune microenvironment remodeling in advanced-stage disease.

Based on the median *CXCL13* expression value, the patient cohort was stratified into two groups: high-expression and low-expression. The solid line shows the estimated survival probability. Dashed lines represent the 95% confidence intervals (lower and upper limits). Survival outcomes were assessed using the Kaplan–Meier estimator, and intergroup differences were evaluated with the log-rank test. A statistically significant prolongation of overall survival was observed in the high-expression group (*p* < 0.05).

*CXCL13* expression levels were contrasted between normal endometrial samples and UCEC tumor specimens. The Wilcoxon rank-sum test was applied for statistical comparison, revealing a marked upregulation of *CXCL13* in tumor tissues (*p* = 0.0031).

*CXCL13* expression levels were examined across UCEC samples of distinct histological grades (G1, G2, and G3). Statistical significance among the three groups was determined using the Kruskal–Wallis rank-sum test, allowing assessment of expression differences by tumor grade.

### 3.9. The Role of CXCL13 as a Putative Biomarker for Immune Activity Within the Tumor Microenvironment

**Figure 22:**
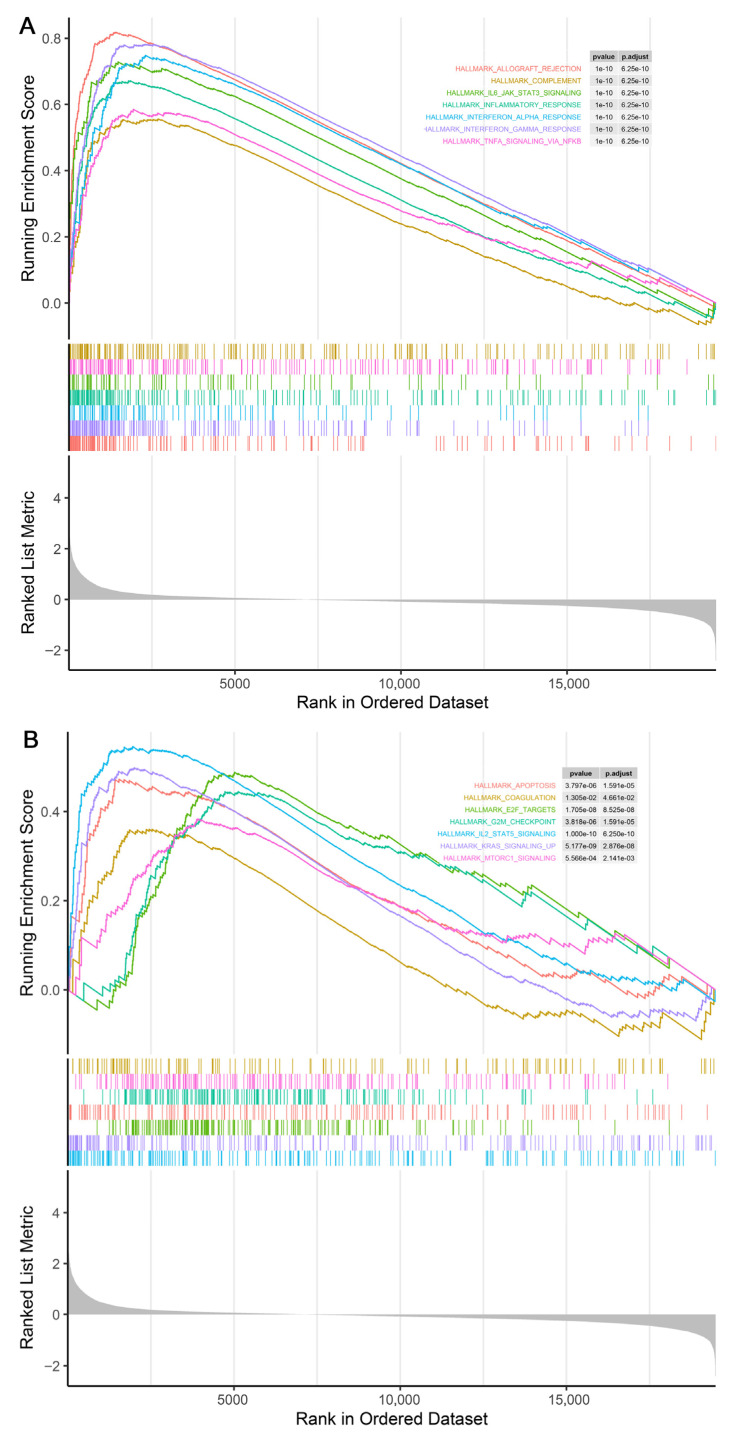
(**A**,**B**) Gene Set Enrichment Analysis (GSEA) of HALLMARK gene sets in the *CXCL13*-high UCEC cohort.

**Figure 23:**
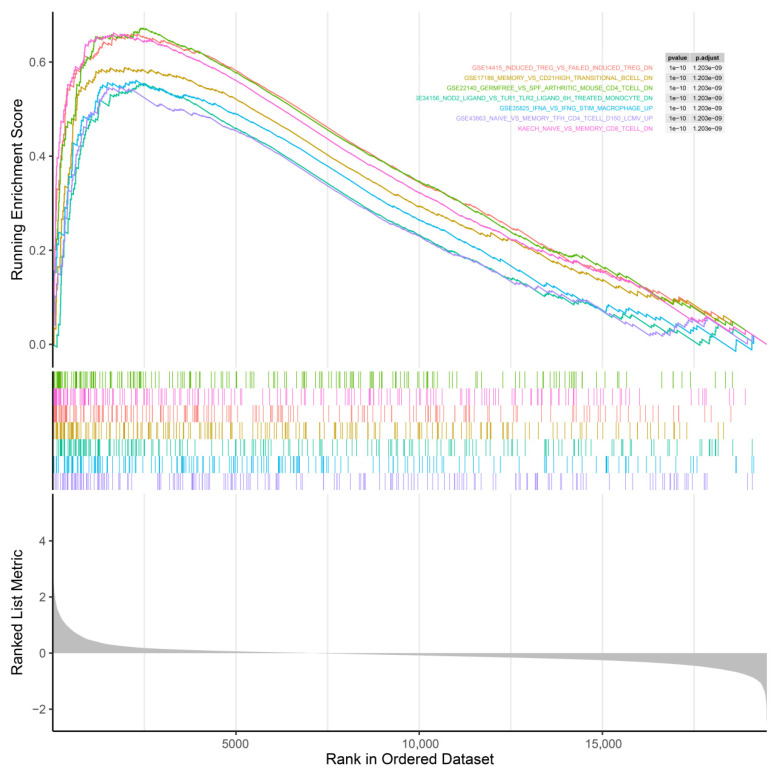
GSEA of the C7 Immune-Related Gene Set in *CXCL13*-high tumors.

**Figure 24:**
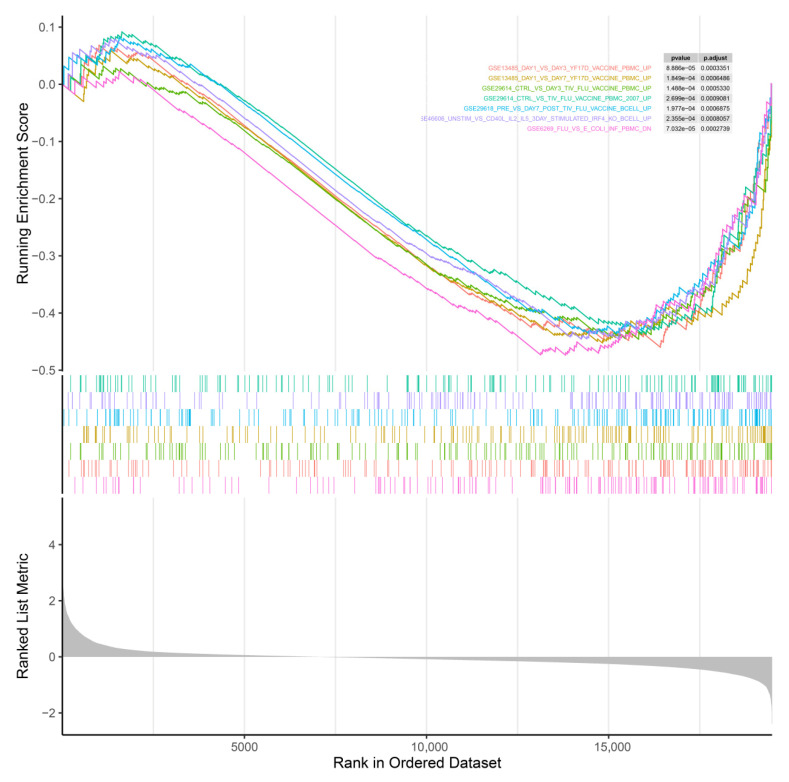
GSEA of the C7 Immune Signature Gene Set in Samples in *CXCL13*-low tumors.

Given the observed associations between *CXCL13* transcript levels, patient survival outcomes, and tumor grade in UCEC, we performed gene set enrichment analysis (GSEA) to delineate its associated functional networks. Patient cohorts were stratified by median *CXCL13* expression. The high-expression group demonstrated pronounced enrichment of immune-related pathways within the Hallmark gene sets, most notably those linked to graft rejection (NES = 3.35, FDR q < 0.001) and interferon-gamma signaling (NES = 3.19, FDR q < 0.001) (Figure 22A,B; Table S4). Complementary analysis using the MSigDB C7 immunologic signature gene sets further corroborated these findings, showing a significant overrepresentation of immune activation pathways in the high-*CXCL13* group (Figure 23; Table S5). In contrast, the low-*CXCL13* group exhibited minimal pathway enrichment (Figure 24; Table S6). These distinct expression profiles suggest that elevated *CXCL13* expression correlates with a phenotypic switch within the TME, transitioning from an immunosuppressive state towards an immunologically active one. When considered alongside its positive correlation with tertiary lymphoid structure (TLS) formation, these findings support the potential utility of *CXCL13* as a dynamic molecular indicator of TME immune status, where its expression level may serve as a surrogate for the local anti-tumor immune response intensity and predict responsiveness to immunotherapy.

Shown here are the GSEA results for Hallmark gene sets, highlighting the top seven most significantly enriched pathways in tumors with high *CXCL13* expression. Each colored curve represents an individual gene set; deflection of the enrichment score (ES) curve to the left of the origin indicates upregulation in the high-expression group, whereas deflection to the right denotes downregulation. Only gene sets meeting the predefined significance criteria (NOM *p* < 0.05, FDR q < 0.06) are displayed, with representative enriched pathways listed.

This panel presents additional GSEA results for Hallmark gene sets, corresponding to the remaining seven significantly enriched pathways in the *CXCL13*-high expression group. As in Figure 22, pathway upregulation or downregulation is inferred from the direction of the ES curve relative to the origin, and only gene sets satisfying NOM *p* < 0.05 and FDR q < 0.06 are shown.

GSEA was performed using the MSigDB C7 collection (immune-related gene sets) to assess pathway enrichment in UCEC samples stratified by *CXCL13* expression. The top seven positively enriched pathways in the high-expression group are displayed (NOM *p* < 0.05, FDR q < 0.05). These findings further underscore the strong association between elevated *CXCL13* expression and the activation of specific immune response programs.

GSEA of the MSigDB C7 collection was similarly conducted for UCEC samples with low *CXCL13* expression. The top seven negatively enriched pathways in this group are shown (NOM *p* < 0.05, FDR q < 0.05), revealing distinct alterations in immune-related pathways associated with low *CXCL13* expression states.

### 3.10. Association Between CXCL13 Expression and Transcriptome-Inferred Immune Cell Composition

**Figure 25:**
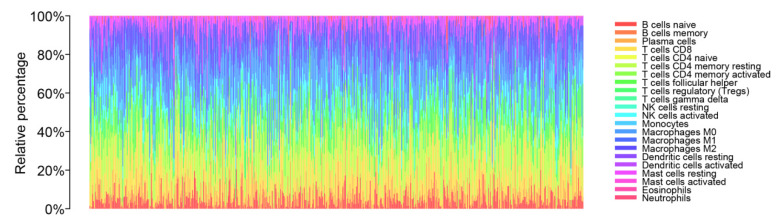
Transcriptome-inferred immune-cell composition across UCEC tumor samples.

**Figure 26:**
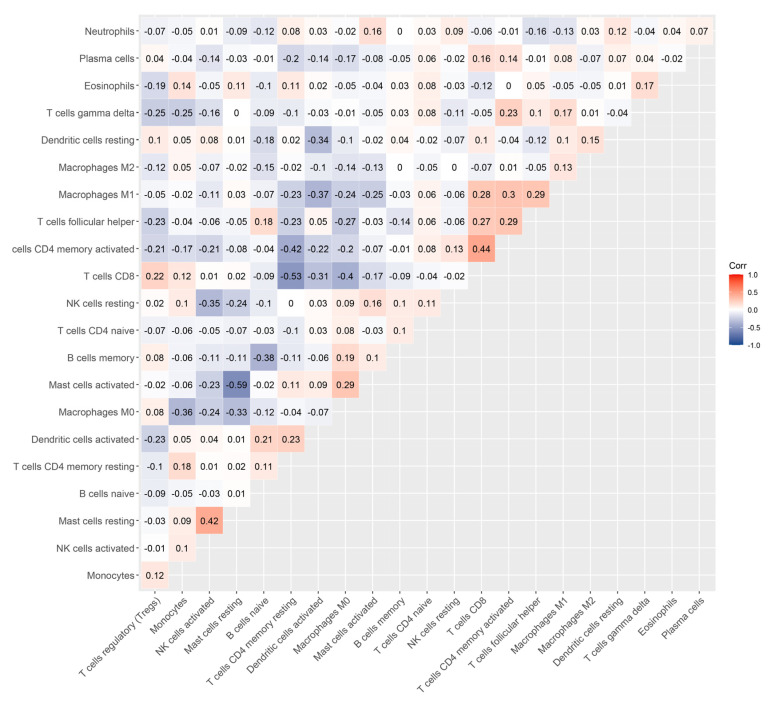
Spearman correlation matrix of transcriptome-inferred immune-cell populations.

**Figure 27:**
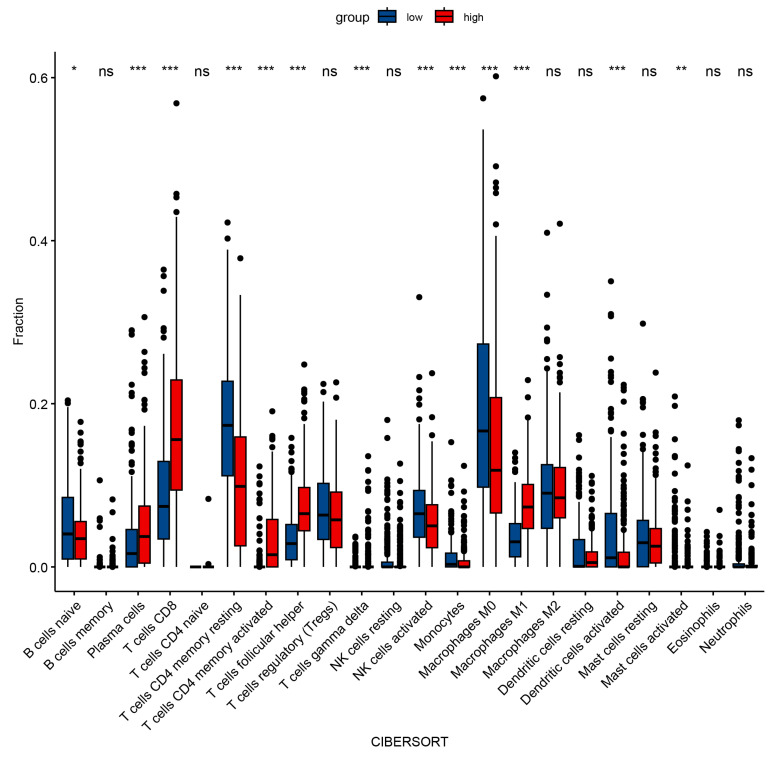
Differential abundance of immune cell subsets according to *CXCL13* expression. Violin plots comparing the relative proportions of 22 immune cell types between UCEC patient groups stratified by median *CXCL13* expression (high vs. low). Statistical significance was evaluated using the Wilcoxon rank-sum test. Violin plots with individual grey dots (*n* = 539). Boxplot elements: median, IQR, and 5th–95th percentiles. Wilcoxon rank-sum test; ns *p* ≥ 0.05, * *p* < 0.05, ** *p* < 0.01, *** *p* < 0.001.

**Figure 28:**
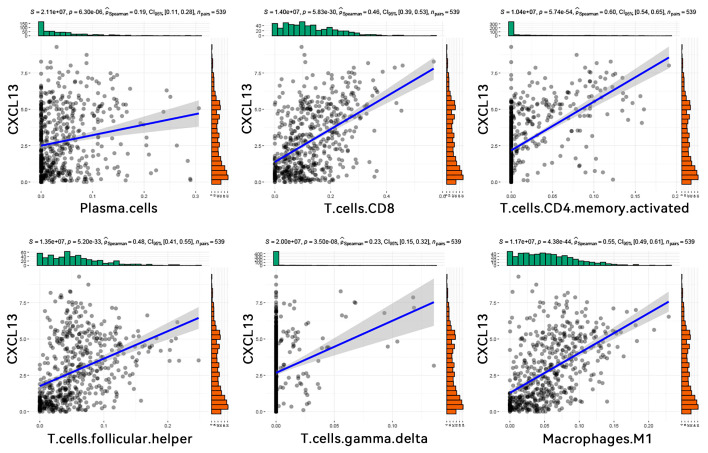
Immune cell subsets positively correlated with *CXCL13* expression.

**Figure 29:**
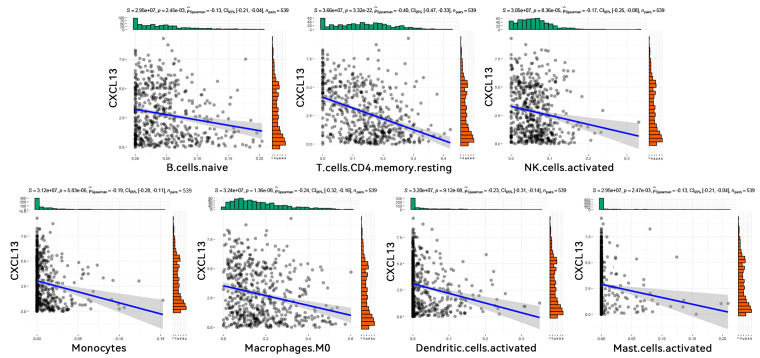
Immune cell subsets negatively correlated with *CXCL13* expression.

To evaluate the association between *CXCL13* expression and transcriptome-inferred immune-cell composition, we applied the CIBERSORT algorithm to estimate the relative fractions of 22 immune cell types across 539 TCGA-UCEC tumor specimens (Figure 25 and Figure 26). Spearman correlation analysis showed that *CXCL13* transcript levels were significantly associated with the estimated abundance of 13 immune cell subsets (*p* < 0.05; Figure 27, Figure S1, Table S7). Positive correlations were observed between *CXCL13* expression and plasma cells (ρ = 0.19), CD8^+^ T cells (ρ = 0.46), activated memory CD4^+^ T cells (ρ = 0.60), follicular helper T cells (ρ = 0.48), γδ T cells (ρ = 0.23), and M1 macrophages (ρ = 0.55) (Figure 28). In contrast, negative correlations were observed with naive B cells (ρ = −0.13), resting memory CD4^+^ T cells (ρ = −0.40), activated natural killer cells (ρ = −0.17), monocytes (ρ = −0.19), M0 macrophages (ρ = −0.24), activated dendritic cells (ρ = −0.23), and activated mast cells (ρ = −0.13) (Figure 29). These CIBERSORT-based results suggest that high *CXCL13* expression is associated with higher estimated abundance of several immune-activating cell subsets and lower estimated abundance of selected resting or immunosuppressive immune-cell subsets.

The relative fractions of 22 immune cell subsets in 539 TCGA-UCEC tumor specimens were estimated using the CIBERSORT algorithm and are shown as a stacked histogram. Each bar represents one tumor sample, and the *y*-axis indicates the estimated proportion of each immune cell type.

This heatmap illustrates pairwise Spearman correlations among 21 CIBERSORT-estimated immune cell subsets. Color intensity reflects the direction and strength of association, and statistically significant correlations are shown according to *p* < 0.05.

Scatter plots depicting significant positive correlations between *CXCL13* transcript levels and the estimated proportions of six immune cell subsets (*p* < 0.05 for each). Linear regression lines (in red) are overlaid to illustrate the trend, with correlation strength quantified using Pearson’s correlation coefficient.

Scatter plots showing significant negative correlations between *CXCL13* expression and the estimated proportions of seven immune cell subsets (*p* < 0.05 for each). Linear regression lines (in red) are provided to visualize the inverse relationship, with correlation strength determined by Pearson’s correlation coefficient. Overall, these CIBERSORT-based results suggest that high *CXCL13* expression is associated with higher estimated abundance of several immune-activating cell subsets and lower estimated abundance of selected immunosuppressive or resting immune-cell subsets.

### 3.11. Cellular Origin of CXCL13: Predominant Expression in Follicular Helper and Exhausted CD8^+^ T Cells

To establish the clinical relevance of single-cell expression patterns, we first evaluated the representativeness of the single-cell dataset (GSE173682) employed in this study. The 11 endometrial cancer specimens in this cohort encompassed FIGO stages I through IV, with a stage distribution of 54.5% stage I, 27.3% stages II–III, and 18.2% stage IV. This distribution did not differ significantly from that of the TCGA multi-omics reference cohort (*n* = 539; *p* = 0.129, chi-square test), confirming that the single-cell data adequately capture the clinical heterogeneity of UCEC.

**Figure 30:**
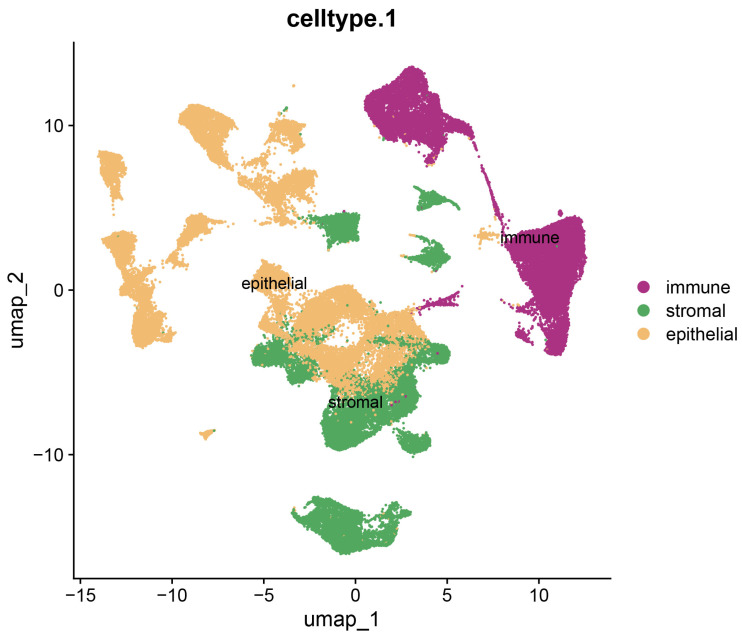
Overview of the UCEC single-cell atlas at the major lineage level.

**Figure 31:**
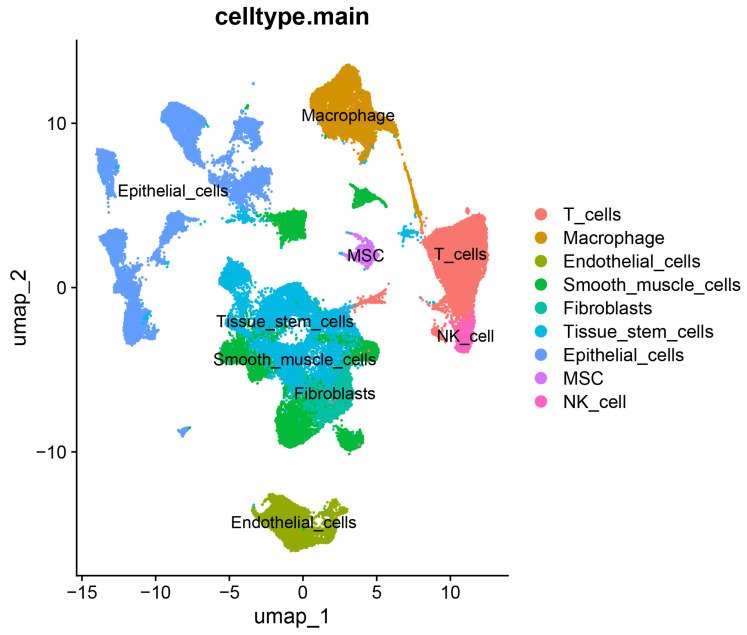
High-resolution single-cell profiling of UCEC.

Leveraging this representative single-cell atlas, we investigated the cellular sources of *CXCL13* within the UCEC tumor microenvironment. The GSE173682 dataset, comprising 92,437 cells, underwent systematic processing including quality filtering, normalization, and batch effect correction via Harmony, followed by clustering and annotation using Seurat v4.3. Following rigorous quality control, 34,312 high-quality cells were retained and categorized into nine major cell lineages (Figure 30 and Figure 31). UMAP visualization revealed marked heterogeneity among immune and stromal compartments, whereas epithelial cells formed a discrete cluster, providing a spatial framework for localizing *CXCL13* expression.

Following quality control, doublet removal, normalization, and Harmony-based batch integration of the GSE173682 dataset, 34,312 high-quality cells were retained. Seurat clustering (resolution = 1) yielded 35 clusters, which were categorized into three major lineages based on canonical marker genes: immune (PTPRC; *n* = 14,207), stromal (MME/PECAM1; *n* = 12,104), and epithelial (EPCAM; *n* = 8001). UMAP projection revealed intermingling of immune and stromal populations, whereas epithelial cells formed a more discrete cluster, reflecting the inherent heterogeneity of the tumor microenvironment. This lineage framework served as the basis for subsequent analyses of *CXCL13* cellular origin and clinical relevance.

After stringent quality control, doublet elimination, normalization, and Harmony integration, 34,312 high-quality cells were subjected to Seurat clustering (resolution = 1), resulting in 35 clusters. Further annotation based on lineage-defining and functional markers identified nine major cell types: T cells (*n* = 9842), macrophages (*n* = 5113), epithelial cells (*n* = 8001), endothelial cells (*n* = 2847), smooth muscle cells (*n* = 2310), fibroblasts (*n* = 2104), tissue stem cells (*n* = 1908), mesenchymal stem cells (*n* = 1002), and natural killer cells (*n* = 1185). UMAP visualization demonstrated substantial intermingling of immune and stromal compartments, with epithelial cells forming a relatively distinct cluster, underscoring the marked heterogeneity of the UCEC tumor microenvironment.

**Figure 32:**
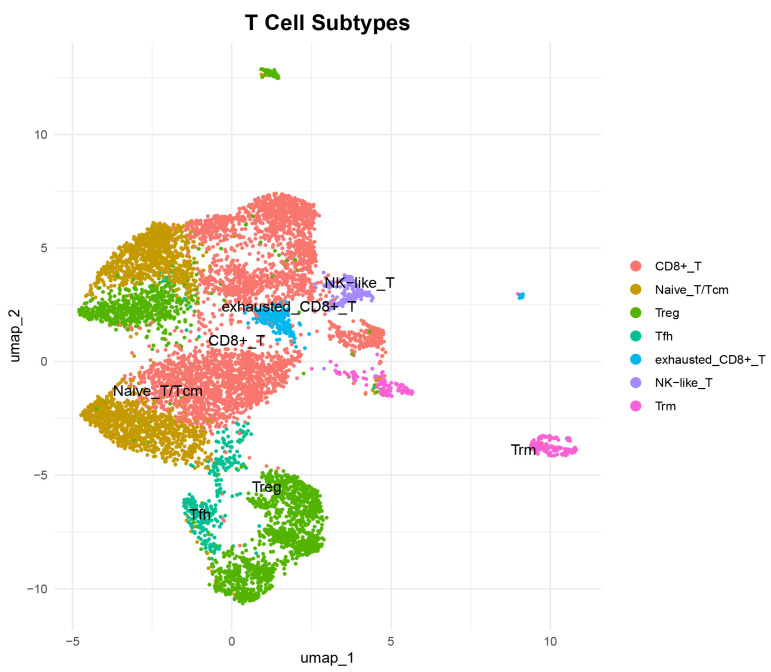
High-resolution mapping of T-cell subsets within the UCEC microenvironment.

**Figure 33:**
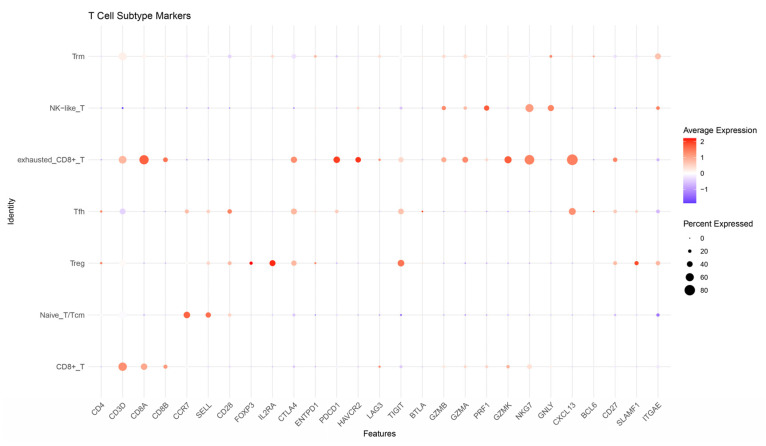
Gene expression signatures of T-cell subsets in the UCEC microenvironment.

**Figure 34:**
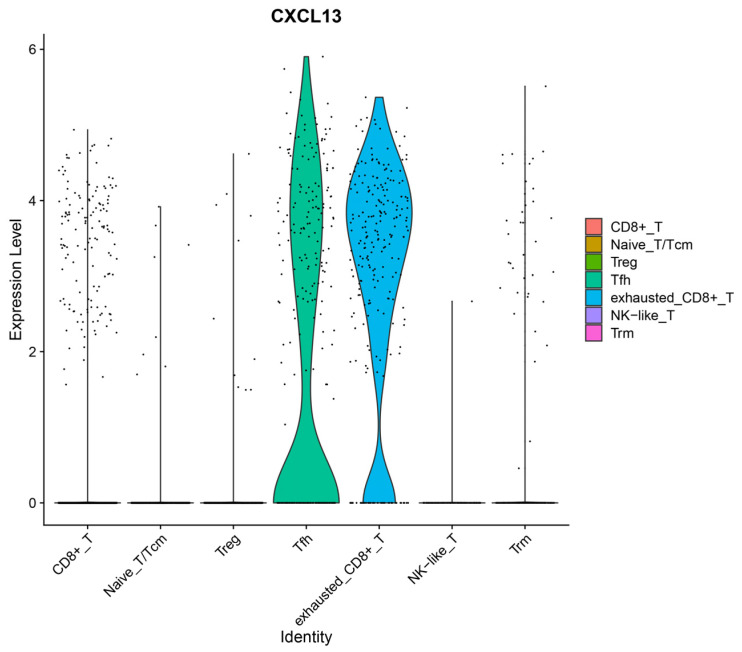
Differential *CXCL13* expression across T-cell subsets in the UCEC microenvironment.

**Figure 35:**
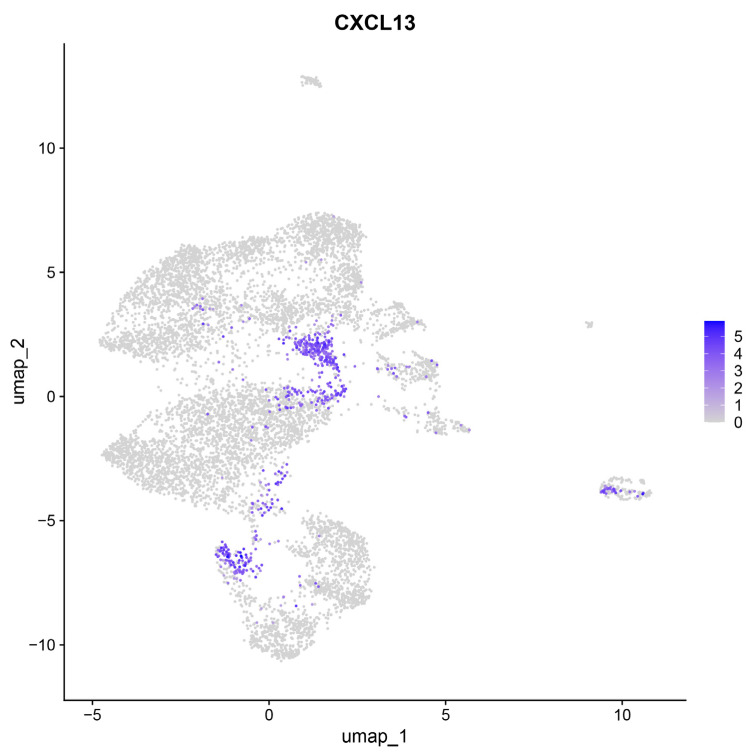
Spatial distribution of *CXCL13* expression across T-cell subsets in the UCEC microenvironment.

To resolve *CXCL13* expression at higher resolution, 9842 T cells were subjected to reclustering (resolution = 0.6), yielding seven distinct functional subsets (Figure 32). Analysis of subset-specific marker genes demonstrated that *CXCL13* expression was significantly enriched in follicular helper T (Tfh) cells and exhausted CD8^+^ T cells, with transcript levels in these populations substantially exceeding those observed in other T-cell subsets (Figure 33 and Figure 34). Dimensional reduction projections further corroborated this pattern, showing *CXCL13* enrichment in regions corresponding to Tfh and exhausted CD8^+^ T cells, alongside strong positive correlation with the canonical Tfh marker *BCL6* and the exhaustion-associated gene *LAG3* (Figure 35).

Following quality filtering, 9842 T cells were subjected to dimensionality reduction (PCA with 30 components followed by UMAP) and clustering at a resolution of 0.6, yielding seven distinct functional subsets. UMAP coordinates revealed that CD8^+^ effector and exhausted CD8^+^ T cells aligned along the umap_1 axis, with central memory/naive T cells positioned above, whereas Treg and Tfh subsets exhibited independent clustering patterns. This organization highlights the pronounced functional polarization of T cells within the tumor microenvironment and provides a high-resolution framework for evaluating *CXCL13* expression across T-cell subsets and its role in immune modulation.

A bubble plot was generated for 9842 reclustered T cells, displaying expression levels (color gradient: blue-white-red) and fraction of expressing cells (bubble size) for 14 functionally relevant genes. Key observations include the following: (1) Tfh cells showed high expression of *CXCL13*, *BCL6*, and *CD27*, consistent with their follicular helper phenotype; (2) exhausted CD8^+^ T cells were enriched for *PDCD1*, *LAG3*, and *TIGIT*, indicative of chronic antigen stimulation; (3) Tregs specifically expressed *FOXP3*, *IL2RA*, and *CTLA4*, reflecting their immunosuppressive capacity; and (4) Trm cells exhibited elevated ITGAE expression, whereas naive/central memory T cells showed higher *CCR7* and *SELL* levels, suggesting distinct residency and migratory characteristics. The selective upregulation of *CXCL13* in Tfh cells supports its role in shaping the immune landscape through lymphocyte chemotaxis.

Violin plots depict the distribution of *CXCL13* expression (RNA-normalized, log1p-transformed) across seven reclustered T-cell subsets (*n* = 9842). *CXCL13* expression demonstrated pronounced subset specificity: Tfh cells exhibited the highest levels, with median expression significantly exceeding that of all other subsets (*p* < 0.001). Moderate expression was observed in exhausted CD8^+^ T cells, whereas naive/Tcm, Treg, NK-like T, and Trm cells showed negligible expression. These findings confirm *CXCL13* as a lineage-associated marker for Tfh cells and suggest its potential involvement in shaping the lymphoid immune profile of UCEC via local chemotactic mechanisms.

Using a UMAP embedding of 9842 reclustered T cells, FeaturePlot was applied to visualize *CXCL13* expression (RNA layer, normalized log1p values). A gray-to-red color gradient (not detected to high expression) revealed predominant enrichment of *CXCL13* in the Tfh cluster (cluster 8), with lower expression in CD8^+^ T-cell clusters (clusters 11 and 16) and absence in naive/Tcm and NK-like T-cell subsets. This distribution pattern indicates that *CXCL13* functions not only as a Tfh lineage marker but may also contribute to reshaping tumor lymphocytic infiltration through local chemotactic activity, providing single-cell evidence for its prognostic and immunoregulatory roles.

**Figure 36:**
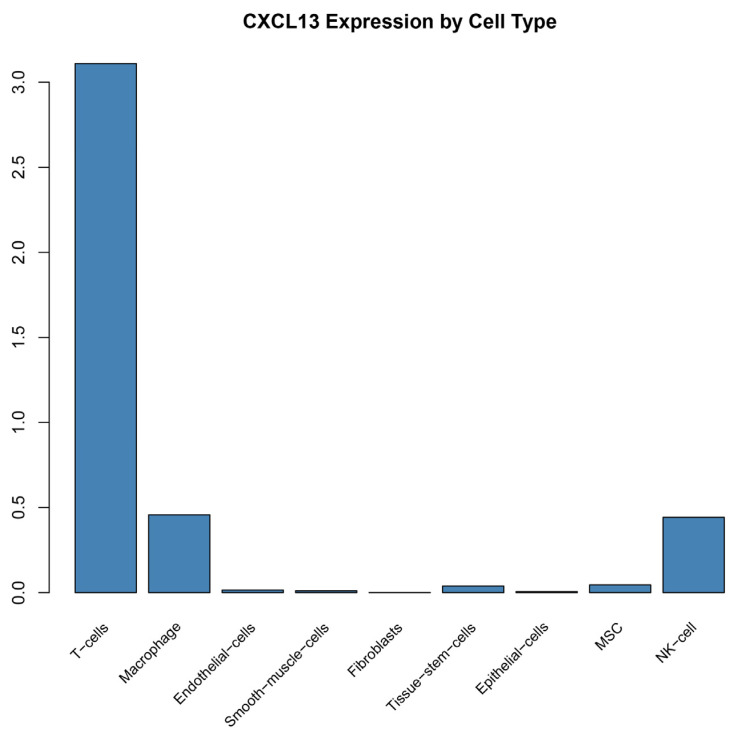
Cell-type-specific *CXCL13* expression in the UCEC single-cell transcriptomic landscape.

**Figure 37:**
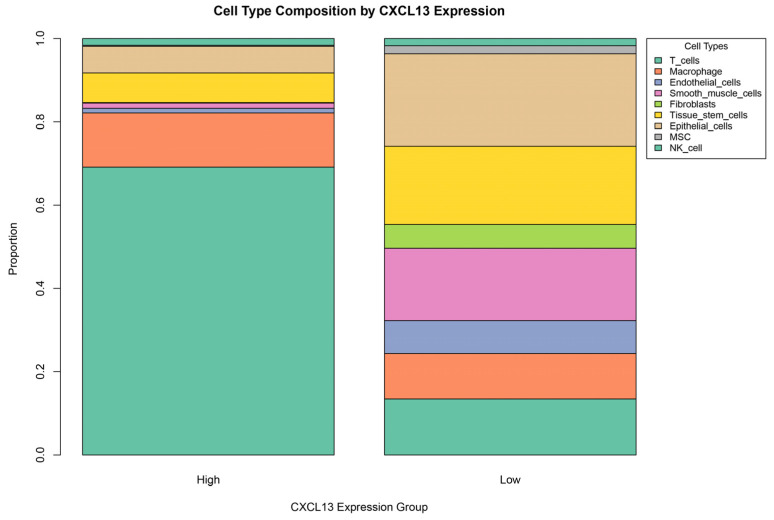
Comparative cell composition of the UCEC tumor microenvironment between groups with high versus low *CXCL13* expression.

**Figure 38:**
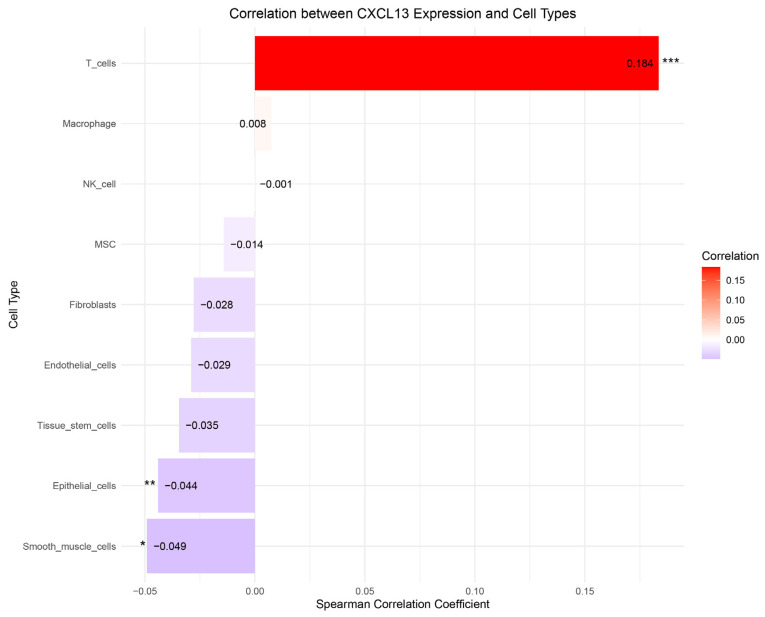
Relationship between *CXCL13* expression levels and immune cell infiltration in the UCEC microenvironment. Spearman’s rank correlation was used to evaluate associations between *CXCL13* transcript abundance and the relative proportions of annotated cell types. A positive correlation was observed between *CXCL13* expression and T-cell abundance, whereas weak negative correlations were observed with selected stromal or epithelial components. These results indicate an association between *CXCL13* expression and a T-cell-enriched transcriptomic profile. Spearman correlation coefficients (ρ) are shown for each major cell type. Asterisks denote statistical significance: * *p* < 0.05, ** *p* < 0.01, *** *p* < 0.001. Only T cells showed a significant positive correlation (ρ = 0.184, *p* < 0.001); all other cell types had *p* ≥ 0.05 (ns, not significant).

Notably, *CXCL13* transcripts were barely detectable in stromal cells (including endothelial cells and fibroblasts) and epithelial cells (Figure 36). Comparative analysis of cellular composition between *CXCL13* expression strata revealed a marked increase in T-cell infiltration in the high-expression group (*p* < 0.05), with the most pronounced gains observed specifically within Tfh and exhausted CD8^+^ T subsets (Figure 37). Spearman correlation analysis further supported a positive association between *CXCL13* expression and overall T-cell abundance (r = 0.184, *p* < 0.001), alongside a weak negative correlation with epithelial cell proportions (r = −0.044) (Figure 38). As a chemokine that engages *CXCR5* to orchestrate B-cell and Tfh recruitment and facilitate tertiary lymphoid structure (TLS) formation, the preferential production of *CXCL13* by Tfh and exhausted CD8^+^ T cells suggests a potential positive feedback loop, wherein these populations may self-amplify or recruit additional T-cell subsets, thereby driving immune remodeling within the tumor microenvironment.

Based on 34,312 high-quality cells, Seurat clustering identified 35 clusters that were annotated into nine major cell types. Average *CXCL13* expression per cell type was calculated using the AverageExpression function and visualized as a bar chart. *CXCL13* expression was mainly observed in immune-cell populations, particularly T cells, while epithelial, endothelial, smooth muscle, fibroblast, and mesenchymal stem cell populations showed relatively low expression. These results suggest immune-cell-associated *CXCL13* expression in the analyzed scRNA-seq dataset.

Cells were divided into high- and low-*CXCL13* expression groups according to median *CXCL13* expression. Stacked bar plots showed differences in the relative proportions of annotated cell types between groups. The high-*CXCL13* group showed a higher relative proportion of T cells than the low-*CXCL13* group. These findings describe cell-composition differences associated with *CXCL13* expression and should not be interpreted as direct evidence of chemokine-mediated cell recruitment.

Taken together, the single-cell analysis suggests that *CXCL13* expression is mainly associated with T-cell subsets, particularly follicular helper T cells and exhausted CD8^+^ T cells, and is linked to a T-cell-enriched transcriptomic profile in the analyzed public scRNA-seq dataset.

## 4. Discussion

By integrating publicly available TCGA transcriptomic data with a public single-cell RNA-sequencing dataset, this study evaluated the prognostic relevance of *CXCL13* and its association with immune microenvironmental features in uterine corpus endometrial carcinoma (UCEC). Our findings suggest that elevated *CXCL13* expression is associated with favorable overall survival and an immune-active tumor microenvironment (TME) profile. In bulk RNA-seq analyses, high *CXCL13* expression was correlated with immune-activation pathways and with CIBERSORT-estimated increases in CD8^+^ T cells, plasma cells, follicular helper T cells, and M1 macrophages. Single-cell analysis further suggested that *CXCL13* expression was mainly associated with follicular helper T cells and exhausted CD8^+^ T cells. These findings support the hypothesis that *CXCL13* may be linked to immune-active TME states in UCEC; however, they should be interpreted as transcriptomic associations rather than direct histological or functional evidence of immune-cell recruitment or TLS formation.

These findings provide an initial outline of *CXCL13*’s immunoregulatory profile at the cellular population level; however, how this chemokine functions within the tissue’s spatial architecture and how it synergizes with other chemokines to promote TLS maturation still requires interpretation in conjunction with morphological evidence. Recent studies have provided key clues for understanding this issue.

### 4.1. Synergistic Mechanisms Involving CXCL13 and TLS Formation

Utilizing immunohistochemistry and spatial transcriptomics on a cohort of 45 endometrial carcinoma samples, Nagase et al. provided the first histological evidence directly linking *CXCL13* expression to TLS formation [20]. Their findings revealed that within the subgroup characterized by high immunogenicity, the density of TLS was markedly elevated, accompanied by a concomitant upregulation of both *CXCL13* and *CXCL9*. Spatial localization analyses further demonstrated that *CXCL13*-positive follicular dendritic cells predominantly resided within the B-cell compartments of TLS, whereas *CXCL9*-positive antigen-presenting cells were concentrated in the T-cell zones. This spatial arrangement suggests a cooperative interplay between these two cell populations in orchestrating an immune-active microenvironment. These observations from previous histological and spatial studies provide a useful biological context for interpreting the immune-active transcriptomic features observed in the present analysis. However, whether *CXCL13*-associated immune signatures in our cohort correspond to mature TLS structures requires direct validation using tissue-based approaches.

Integrating these observations with the single-cell transcriptomic data presented here—which identified Tfh and exhausted CD8^+^ T cells as the principal sources of *CXCL13*—we propose that the “*CXCL13*-*CXCL9* axis” plays a pivotal role in immune remodeling within UCEC. In this model, *CXCL13* derived from Tfh and exhausted CD8^+^ T cells recruits B cells and additional Tfh cells into the germinal centers of TLS via *CXCR5* binding. Concurrently, *CXCL9* facilitates the recruitment of effector T cells and antigen-presenting cells through its receptor *CXCR3*. The spatiotemporal coordination of these two chemokine signals facilitates the transition of TLS from nascent lymphocyte aggregates into functionally mature immune structures. This mechanistic framework not only accounts for the observed positive correlation between *CXCL13* expression and TLS formation, but also provides a more detailed molecular understanding of its role in promoting immune activation within the tumor microenvironment.

### 4.2. Comparative Analysis with Other Tumor Types

The identification of the aforementioned synergistic mechanisms prompts us to further consider whether the *CXCL13*-mediated regulation of TLS is a universal phenomenon across different tumor types. To this end, we conducted a systematic comparison of our findings with recent studies on *CXCL13* in other tumor types.

Work by Chen Z. and colleagues in colorectal cancer revealed that *CXCL13*^+^ CD8^+^ T cells and CD4^+^ Tfh cells collaboratively orchestrate TLS functions via CD40-CD40L signaling, thereby affecting the clinical response to immune checkpoint inhibitors [36]. This pattern of intercellular communication closely parallels our observation of *CXCL13* co-expression by Tfh and exhausted CD8^+^ T cells, implying that *CXCL13* may contribute to TLS assembly through evolutionarily conserved cellular networks. Separately, single-cell transcriptomic profiling of cutaneous squamous cell carcinoma by Chen Y. and colleagues identified T cells and fibroblasts as the predominant cellular sources of *CXCL13*, with its transcript levels exhibiting a strong positive association with TLS density [37]. Although this study identified fibroblasts as an additional source—in contrast to our finding that *CXCL13* in UCEC is secreted almost exclusively by immune cells—both investigations collectively reinforce the concept that *CXCL13* acts as a critical driver of TLS formation.

These cross-cancer comparisons indicate that while the function of *CXCL13* exhibits some tissue specificity (e.g., differences in cellular sources), its fundamental role in promoting TLS formation and reshaping the immune microenvironment is highly conserved across different tumors. This conservation provides a theoretical basis for developing pan-cancer immunotherapy strategies targeting *CXCL13*.

### 4.3. Clinical Significance and Translational Prospects

From a clinical perspective, the association between high *CXCL13* expression, favorable prognosis, and immune-active transcriptomic features suggests that *CXCL13* may have value as a biomarker candidate for immune microenvironment stratification in UCEC. However, because this study did not include an independent prospective cohort, immunotherapy-response data, or tissue-based validation, the predictive value of *CXCL13* for immune checkpoint blockade remains uncertain. Future studies should evaluate *CXCL13* in clinically annotated cohorts with treatment-response information and should combine transcriptomic analysis with histological or spatial validation before considering therapeutic translation.

### 4.4. Relationship with Previous GWAS Findings in Uterine Cancer

Previous GWAS of uterine or endometrial cancer have mainly focused on inherited susceptibility loci and germline genetic risk, whereas the present study analyzed tumor transcriptomic profiles, immune-cell deconvolution, and single-cell expression patterns. Therefore, the current findings do not directly identify inherited risk variants for UCEC. Instead, they provide expression-level and microenvironment-level evidence suggesting that *CXCL13* is associated with immune-active tumor states and favorable prognosis. The relationship between our results and GWAS findings should therefore be interpreted as complementary rather than directly overlapping: GWAS can identify genetic loci that influence cancer susceptibility, while transcriptomic and single-cell analyses can reveal downstream immune phenotypes within established tumors. Future integrative analyses combining GWAS loci, expression quantitative trait loci, tumor RNA-seq, and single-cell or spatial transcriptomic data will be needed to determine whether inherited susceptibility variants influence *CXCL13*-related immune programs in UCEC.

### 4.5. Future Directions and Translational Perspectives

In summary, this work supports *CXCL13* as a candidate prognostic indicator and an immune microenvironment-associated gene in UCEC. However, the present findings are based on public transcriptomic and single-cell datasets and therefore remain primarily correlative. Further studies are required to determine whether *CXCL13* directly regulates immune-cell recruitment, TLS-associated organization, and antitumor immune activity.

First, the molecular mechanisms underlying *CXCL13*-associated immune remodeling require functional validation. Future studies may use *CXCL13* gain- and loss-of-function models, UCEC organoid co-culture systems, and in vivo tumor models to examine its potential effects on follicular helper T-cell differentiation, CD8^+^ T-cell function, and TLS-related immune organization. Spatial transcriptomics, multiplex immunofluorescence, and RNAscope-based approaches could further clarify whether *CXCL13*-high regions colocalize with T-cell/B-cell-enriched immune niches, interferon-related signaling, and metabolic remodeling pathways such as *JAK-STAT* activation and glycolytic programs involving *HK2* and *LDHA*.

Second, the clinical value of *CXCL13* should be tested in independent, well-annotated UCEC cohorts. Multicenter retrospective and prospective studies are needed to evaluate whether *CXCL13* expression can improve prognostic stratification when combined with clinicopathological variables, molecular subtype, circulating tumor DNA features, or imaging-derived parameters. Tissue-based assays targeting *CXCL13* and Tfh-associated markers such as *BCL6*, *CXCR5*, and *PDCD1* may also help determine whether *CXCL13*-associated immune states can be assessed in routine or translational pathology settings.

Third, interdisciplinary approaches may provide a more comprehensive understanding of *CXCL13*-related tumor microenvironment dynamics. Integration of transcriptomics, epigenomics, proteomics, spatial profiling, and artificial intelligence-based modeling could help construct dynamic immune microenvironment scores and predict treatment response. Patient-derived organoids, 3D tumor models, and metabolic flux assays may further clarify whether *CXCL13*-associated immune activation interacts with tumor metabolic states and whether metabolic intervention could enhance immune responsiveness.

Finally, the broader relevance of *CXCL13* across gynecological and hormone-related malignancies remains to be explored. Comparative analyses of single-cell and spatial transcriptomic datasets from UCEC, high-grade serous ovarian cancer, cervical cancer, and breast cancer may help distinguish tissue-specific from conserved *CXCL13*-associated immune programs. Particular attention should be paid to whether these patterns differ across UCEC molecular subtypes, including POLE-hypermutated and TP53-abnormal tumors. Such studies would provide a stronger biological basis for evaluating *CXCL13* as a biomarker candidate, while therapeutic translation should remain dependent on rigorous tissue-based and functional validation.

## 5. Conclusions

In this study, we integrated public TCGA-UCEC transcriptomic data with a public single-cell RNA-sequencing dataset to evaluate the prognostic relevance and immune microenvironment-associated features of *CXCL13* in UCEC. High *CXCL13* expression was associated with favorable overall survival, enrichment of immune-activation pathways, and increased CIBERSORT-estimated fractions of selected immune-cell subsets, including CD8^+^ T cells, plasma cells, follicular helper T cells, and M1 macrophages. Single-cell analysis suggested that *CXCL13* expression was mainly associated with follicular helper T cells and exhausted CD8^+^ T cells. These findings support *CXCL13* as a prognosis-associated immune microenvironment marker in UCEC.

## 6. Limitations

This study has several limitations. First, all analyses were based on publicly available datasets, and no in-house patient specimens or newly generated sequencing data were included. Second, immune-cell proportions derived from CIBERSORT should be interpreted as transcriptome-inferred estimates rather than histologically or immunohistochemically measured cell counts. Third, TLSs were not directly identified by hematoxylin-eosin staining, immunohistochemistry, multiplex immunofluorescence, or spatial transcriptomics in this study; therefore, TLS-related interpretations remain based on transcriptional features and prior literature. Fourth, the public scRNA-seq dataset used here has a limited sample size and includes heterogeneous tumor contexts, which may restrict its representativeness for the broader UCEC population. Fifth, the analyses are primarily correlative and cannot establish a causal role for *CXCL13* in immune-cell recruitment, TLS formation, or treatment response. Finally, independent validation cohorts, tissue-based spatial analyses, and functional perturbation experiments are required to confirm the biological and clinical significance of *CXCL13* in UCEC.

## Figures and Tables

**Table 1:** Summary of clinical characteristics and single-cell metadata for each tumor.

Patient	GEO Sample Number	Cancer Type	Tumor Site	Histology	Stage	Age	BMI	scATAC-seq Cells	scRNA-seq Cells
Patient 1	GSM5276933	Endometrial	Endometrium	Endometrioid	IA	70	39.89	6348 (6649)	5279 (5697)
Patient 2	GSM5276934	Endometrial	Endometrium	Endometrioid	IA	70	30.57	7248 (6658)	7277 (7963)
Patient 3	GSM5276935	Endometrial	Endometrium	Endometrioid	IA	70	38.55	4165 (7241)	4974 (6054)
Patient 4	GSM5276936	Endometrial	Endometrium	Endometrioid	IA	49	55.29	7597 (7917)	7413 (8110)
Patient 5	GSM5276937	Endometrial	Endometrium	Endometrioid	IA	62	49.44	6797 (7881)	7291 (8403)
Patient 6	GSM5276938	Endometrial	Ovary	Serous	IIIA	74	29.94	6643 (2351)	6866 (8009)
Patient 7	GSM5276939	Ovarian	Ovary	Endometrioid	IA	76	34.85	5924 (7107)	6454 (8295)
Patient 8	GSM5276940	Ovarian	Ovary	HGSOC	IIB	61	22.13	8014 (7898)	7454 (8181)
Patient 9	GSM5276941	Ovarian	Ovary	HGSOC	IIIC	59	22.37	9670 (9942)	6192 (6939)
Patient 10	GSM5276942	Ovarian	Ovary	Carcinosarcoma	IVB	69	23.72	4439 (8977)	7663 (8984)
Patient 11	GSM5276943	Gastric	Ovary	GIST	IV	59	33.96	7776 (11,066)	8660 (10,094)

**Table 2:** Baseline clinicopathological characteristics of TCGA-UCEC patients stratified by *CXCL13*.

Characteristic	Total	*CXCL13*-High	*CXCL13*-Low	*p*-Value
No. of patients	539	270	269	—
*CXCL13* expression, median IQR	2.415 (0.847–4.558)	4.558 (3.423–5.509)	0.842 (0.393–1.485)	<0.001
Age at diagnosis, years, median IQR	64.0 (57.0–71.0)	63.0 (56.0–70.5)	64.0 (58.0–72.0)	0.101
FIGO stage, *n* %	*n* = 466	*n* = 239	*n* = 227	0.466
Stage I	303 65.0	161 67.4	142 62.6	
Stage II	42 9.0	23 9.6	19 8.4	
Stage III	101 21.7	47 19.7	54 23.8	
Stage IV	20 4.3	8 3.3	12 5.3	
Residual disease, *n* %	*n* = 390	*n* = 197	*n* = 193	0.336
R0	327 83.8	167 84.8	160 82.9	
R1	17 4.4	11 5.6	6 3.1	
R2	12 3.1	4 2.0	8 4.1	
RX	34 8.7	15 7.6	19 9.8	
Tumor grade, *n* %	*n* = 463	*n* = 239	*n* = 224	0.005
G1	92 19.9	42 17.6	50 22.3	
G2	106 22.9	44 18.4	62 27.7	
G3	255 55.1	150 62.8	105 46.9	
High Grade	10 2.2	3 1.3	7 3.1	
Histological subtype, *n* %	*n* = 366	*n* = 173	*n* = 193	0.654
Endometrioid	300 82.0	145 83.8	155 80.3	
Serous	53 14.5	22 12.7	31 16.1	
Mixed	13 3.6	6 3.5	7 3.6	
Vital status, *n* %	*n* = 364	*n* = 171	*n* = 193	0.102
Living	325 89.3	158 92.4	167 86.5	
Deceased	39 10.7	13 7.6	26 13.5	
Integrative molecular cluster, *n* %	*n* = 366	*n* = 173	*n* = 193	<0.001
CN high	59 16.1	22 12.7	37 19.2	
CN low	90 24.6	21 12.1	69 35.8	
MSI	65 17.8	42 24.3	23 11.9	
POLE	17 4.6	13 7.5	4 2.1	
Not assigned	135 36.9	75 43.4	60 31.1	
Overall survival time, days, median IQR	712.5 (385.2–1222.2)	714.0 (351.5–1350.5)	709.0 (426.0–1141.0)	0.768

Note: Data are presented as median IQR for continuous variables and *n* % for categorical variables. Patients were divided into high- and low-*CXCL13* expression groups according to the median *CXCL13* expression value. Missing or unknown values were excluded from percentage calculation and statistical testing. Continuous variables were compared using the Wilcoxon rank-sum test, and categorical variables were compared using the chi-square test. FIGO, International Federation of Gynecology and Obstetrics; MSI, microsatellite instability; CN, copy-number.

## Data Availability

The original data presented in the study are publicly available. These data can be found here: TCGA database and GEO database under accession number GSE173682. The source code for the explainable machine learning pipeline is available in the following GitHub repository: https://github.com/Yiwen234/UCEC-CXCL13-Multi-omics-Dataset (accessed on 13 May 2026).

## Supplementary Materials

The following supporting information can be downloaded at: https://www.mdpi.com/article/10.3390/biology15130987/s1, Tables S1–S7, Figures S1–S6, Analysis Parameters and Software Versions, Key analysis code and Data Availability Statement.
