# Supplementary material for: CXCL13 as a Prognostic Biomarker and Immune Microenvironment-Associated Gene in Endometrial Carcinoma: A Multi-Omics Investigation
Source: Biology (Basel). 2026 Jun 23;15(13):987. doi: 10.3390/biology15130987 (PMC13359461; doi:10.3390/biology15130987)
Supplement: Supplementary file 1 [file biology-15-00987-s001.zip › biology-4288514-supplementary.pdf]

Appendix S Supplementary Table

Table S1 The differential expression of CXCL13 in normal and tumor tissues (Wilcoxon test detailed results)

groups	sample size	CXCL13 expression value	mean	median	standard deviation	Wilcoxon test
normal tissue	35	See the list below for details.	1.82	0.98	1.91	p = 0.0031
tumors	539	See the list below for details.	2.81	2.41	2.17	

Note : The mean, median and standard deviation in the table are calculated based on the original data. The data in this table is used to generate the text graph 20.

Table S2 Normal tissue samples (n = 35) CXCL13 expression values ($\log_2$ (FPKM + 1))

sample ID	expression value
TCGA-AX-A05Y-11A	4.40
TCGA-BK-A13C-11A	0.30
TCGA-FL-A1YV-11A	4.63
TCGA-AX-A0J0-11A	4.02
TCGA-E6-A1M0-11A	0.29
TCGA-DI-A2QU-11A	0.25
TCGA-DI-A2QY-11A	2.22
TCGA-AX-A2H8-11A	1.35
TCGA-AX-A2HD-11A	0.75
TCGA-FL-A1YU-11A	0.98
TCGA-BG-A2AD-11A	2.73
TCGA-BG-A3EW-11A	1.30
TCGA-AX-A2HA-11A	4.88
TCGA-FL-A1YG-11A	0.98
TCGA-FL-A1YN-11A	2.13
TCGA-FL-A3WE-11A	0.94
TCGA-FL-A1YI-11A	1.75
TCGA-AX-A0IZ-11A	0.00
TCGA-FL-A1YH-11A	8.03
TCGA-AX-A2HC-11A	3.55
TCGA-FL-A1YQ-11A	4.16
TCGA-BK-A0CB-11A	0.38
TCGA-FL-A1YL-11A	0.13
TCGA-AJ-A2QL-11A	0.16
TCGA-BG-A3PP-11A	0.72
TCGA-AJ-A3NC-11A	0.63
TCGA-AJ-A3NE-11A	1.20
TCGA-AJ-A3NH-11A	0.29
TCGA-FL-A1YT-11A	3.46
TCGA-AX-A1CF-11A	0.50
TCGA-BK-A4ZD-11A	2.23
TCGA-AX-A1CK-11A	0.00
TCGA-FL-A1YF-11A	4.23
TCGA-FL-A1YM-11A	0.00
TCGA-AX-A1CI-11A	0.00

Note : The data in this table is used to generate the text graph 20.

Table S3 Tumor tissue samples (n = 539) CXCL13 expression value ($\log_2$ (FPKM + 1)) Example (sorted by expression level)

sample ID（High expression Top10）	Express-ion value	sample ID（Near the median expression）	Express-ion value	sample ID（Low expression of Bottom10）	Express-ion value
TCGA-EY-A1GE-01A	9.29	TCGA-D1-A102-01A	2.45	TCGA-BG-A0MT-01A	0.00
TCGA-BS-A0U7-01A	8.74	TCGA-D1-A16B-01A	2.45	TCGA-D1-A165-01A	0.00
TCGA-BS-A0TA-01A	8.28	TCGA-BS-A0V7-01A	2.42	TCGA-AX-A1C7-01A	0.00
TCGA-D1-A17A-01A	8.25	TCGA-A5-A2K4-01A	2.42	TCGA-D1-A16Y-01A	0.00
TCGA-A5-A2K3-01A	8.18	TCGA-FI-A2EX-01A	2.41	TCGA-BG-A0W1-01A	0.00
TCGA-AX-A3FT-01A	8.01	TCGA-A5-A3LO-01A	2.40	TCGA-D1-A17R-01A	0.00
TCGA-A5-A0G1-01A	7.87	TCGA-FI-A2F8-01A	2.37	TCGA-BG-A0MG-01A	0.00
TCGA-AX-A06H-01A	7.86	TCGA-EY-A1GD-01A	2.33	TCGA-AP-A0LJ-01A	0.00
TCGA-AP-A059-01A	7.59	TCGA-D1-A0ZV-01A	2.33	TCGA-BG-A0MI-01A	0.00
TCGA-B5-A5OC-01A	7.54	TCGA-BG-A0MO-01A	2.30	TCGA-BG-A0YV-01A	0.00

Note : Here are 10 representative samples of the highest, lowest, and intermediate regions. The expression values of 539 samples in the complete data set are shown in the Github online data set (https://github.com/Yiwen234/CXCL13-/blob/main/CXCL13 normal tumor differential expression.xlsx). The data of this table is used to generate the text graph 20.

Table S4 Complete results of GSEA enrichment analysis (Hallmark gene set)

gene set	NES	FDR q value	NOM p value
HALLMARK_INTERFERON_GAMMA_RESPONSE	3.194	$6.25e^{-10}$	$1.00e^{-10}$
HALLMARK_INTERFERON_ALPHA_RESPONSE	2.787	$6.25e^{-10}$	$1.00e^{-10}$
HALLMARK_INFLAMMATORY_RESPONSE	2.744	$6.25e^{-10}$	$1.00e^{-10}$
HALLMARK_IL6_JAK_STAT3_SIGNALING	2.693	$6.25e^{-10}$	$1.00e^{-10}$
HALLMARK_TNFA_SIGNALING_VIA_NFKB	2.392	$6.25e^{-10}$	$1.00e^{-10}$
HALLMARK_COMPLEMENT	2.274	$6.25e^{-10}$	$1.00e^{-10}$
HALLMARK_IL2_STAT5_SIGNALING	2.231	$6.25e^{-10}$	$1.00e^{-10}$
HALLMARK_KRAS_SIGNALING_UP	2.036	$2.88e^{-08}$	$5.18e^{-09}$
HALLMARK_E2F_TARGETS	1.998	$8.52e^{-08}$	$1.70e^{-08}$
HALLMARK_APOPTOSIS	1.876	$1.59e^{-05}$	$3.80e^{-06}$
HALLMARK_G2M_CHECKPOINT	1.811	$1.59e^{-05}$	$3.82e^{-06}$
HALLMARK_MTORC1_SIGNALING	1.567	0.0021	0.0006
HALLMARK_COAGULATION	1.393	0.0466	0.0131

Note : The data in this table are used to generate Figure 22 and Figure 23.

Table S5 Positive enrichment results of GSEA enrichment analysis (C7 immune gene set)

gene set	NES	FDR q value	NOM p value
GSE7509_UNSTIM_VS_IFNA_STIM_IMMATURE_DC_DN	3.226	$1.20e^{-09}$	$1.00e^{-10}$
GSE7218_UNSTIM_VS_ANTIGEN_STIM_THROUGH_IGG_BCELL_DN	3.214	$1.20e^{-09}$	$1.00e^{-10}$
GSE19888_ADENOSINE_A3R_INH_PRETREAT_AND_ACT_BY_A3R_VS_TCELL_MEMBRANES_ACT_MAST_CELL_UP	3.192	$1.20e^{-09}$	$1.00e^{-10}$
GSE3039_NKT_CELL_VS_ALPHAALPHA_CD8_TCELL_DN	3.099	$1.20e^{-09}$	$1.00e^{-10}$
GSE2935_UV_INACTIVATED_VS_LIVE_SENDAI_VIRUS_INF_MACROPHAGE_DN	3.083	$1.20e^{-09}$	$1.00e^{-10}$
GSE3565_CTRL_VS_LPS_INJECTED_DUSP1_KO_SPLENOCYTES_UP	3.059	$1.20e^{-09}$	$1.00e^{-10}$
GSE13485_DAY1_VS_DAY7_YF17D_VACCINE_PBMC_DN	3.047	$1.20e^{-09}$	$1.00e^{-10}$

Note : The highest positive enrichment of 7 representative samples is shown here. For the enrichment analysis of 2350 samples in the complete data set, see the Github online data set (https://github.com/Yiwen234/CXCL13-/blob/main/c7gsea_results.xlsx)). The data in this table is used to generate the text figure 24.

Table S6 Negative enrichment results of GSEA enrichment analysis (C7 immune gene set)

gene set	NES	FDR q value	NOM p value
GSE19825_NAIVE_VS_DAY3_EFF_CD8_TCELL_UP	- 1.905	$3.03e^{-07}$	$3.94e^{-08}$
GSE6259_DEC205_POS_DC_VS_BCELL_UP	- 1.898	$2.18e^{-06}$	$3.40e^{-07}$
GSE6269_FLU_VS_E_COLI_INF_PBMC_DN	- 1.716	0.0003	$7.03e^{-05}$
GSE13485_DAY1_VS_DAY3_YF17D_VACCINE_PBMC_UP	- 1.681	0.0003	$8.89e^{-05}$
GSE29614_CTRL_VS_DAY3_TIV_FLU_VACCINE_PBMC_UP	- 1.641	0.0005	0.0001
GSE13485_DAY1_VS_DAY7_YF17D_VACCINE_PBMC_UP	- 1.654	0.0006	0.0002
GSE29618_PRE_VS_DAY7_POST_TIV_FLU_VACCINE_BCELL_UP	- 1.630	0.0007	0.0002

Note : Seven representative samples with the highest negative enrichment are shown here. For the enrichment analysis of 2350 samples in the complete data set, see the Github online data set (https://github.com/Yiwen234/CXCL13-/blob/main/c7gsea_results.xlsx)). The data of this table is used to generate the text figure 25.

Table S7 : Correlation analysis between CXCL13 expression and 22 TICs abundanceSome data were analyzed.

Sample ID	TCGA-B5-A0K0-01A	TCGA-AJ-A23O-01A	TCGA-BS-A0VI-01A	TCGA-A5-A2K5-01A	TCGA-D1-A0ZS-01A	TCGA-D1-A160-01A
B cells naive	0.004	0.069	0.053	0.016	0.056	0.041
B cells memory	0.000	0.000	0.000	0.000	0.000	0.000
Plasma cells	0.000	0.142	0.027	0.021	0.012	0.043
T cells CD8	0.321	0.194	0.037	0.190	0.061	0.187
T cells CD4 naive	0.000	0.000	0.000	0.000	0.000	0.000
T cells CD4 memory resting	0.114	0.100	0.170	0.000	0.030	0.131
T cells CD4 memory activated	0.025	0.027	0.000	0.000	0.000	0.032
T cells follicular helper	0.146	0.119	0.004	0.133	0.008	0.110
T cells regulatory (Tregs)	0.117	0.091	0.050	0.072	0.102	0.091
T cells gamma delta	0.000	0.000	0.000	0.000	0.000	0.000
NK cells resting	0.073	0.038	0.000	0.000	0.023	0.000
NK cells activated	0.000	0.014	0.064	0.089	0.000	0.022
Monocytes	0.002	0.030	0.000	0.019	0.000	0.007
Macrophages M0	0.027	0.031	0.487	0.158	0.491	0.162
Macrophages M1	0.064	0.062	0.000	0.106	0.021	0.072
Macrophages M2	0.072	0.061	0.000	0.147	0.085	0.070
Dendritic cells resting	0.021	0.000	0.000	0.004	0.000	0.007
Dendritic cells activated	0.000	0.010	0.104	0.000	0.032	0.000
Mast cells resting	0.016	0.014	0.004	0.043	0.000	0.000
Mast cells activated	0.000	0.000	0.000	0.000	0.070	0.023
Eosinophils	0.000	0.000	0.000	0.000	0.000	0.000
Neutrophils	0.000	0.000	0.000	0.000	0.008	0.000
P-value	0.010	0.010	0.010	0.010	0.010	0.010
Spearman correlation coefficient (ρ)	0.305	0.312	0.312	0.307	0.308	0.316
RMSE	0.984	0.958	0.984	0.965	0.992	0.959

Sample ID	TCGA-BG-A0M7-01A	TCGA-D1-A17F-01A	TCGA-BG-A0MO-01A	TCGA-A5-A0R7-01A	TCGA-AP-A0LP-01A	TCGA-D1-A17S-01A
B cells naive	0.026	0.006	0.023	0.026	0.002	0.027
B cells memory	0.000	0.000	0.000	0.000	0.000	0.000
Plasma cells	0.073	0.039	0.022	0.041	0.000	0.018
T cells CD8	0.113	0.195	0.131	0.216	0.088	0.025
T cells CD4 naive	0.000	0.000	0.000	0.000	0.000	0.000
T cells CD4 memory resting	0.175	0.074	0.074	0.256	0.238	0.156
T cells CD4 memory activated	0.000	0.000	0.020	0.007	0.000	0.000
T cells follicular helper	0.000	0.102	0.045	0.025	0.015	0.002
T cells regulatory (Tregs)	0.100	0.161	0.151	0.134	0.113	0.094
T cells gamma delta	0.000	0.000	0.000	0.000	0.000	0.000
NK cells resting	0.001	0.000	0.000	0.022	0.000	0.009
NK cells activated	0.026	0.058	0.024	0.000	0.066	0.023
Monocytes	0.000	0.002	0.000	0.018	0.013	0.000
Macrophages M0	0.357	0.137	0.221	0.129	0.162	0.471
Macrophages M1	0.036	0.034	0.053	0.046	0.096	0.025
Macrophages M2	0.094	0.121	0.173	0.045	0.152	0.118
Dendritic cells resting	0.000	0.047	0.052	0.000	0.032	0.000
Dendritic cells activated	0.000	0.000	0.000	0.036	0.000	0.000
Mast cells resting	0.000	0.022	0.011	0.000	0.022	0.033
Mast cells activated	0.000	0.000	0.000	0.000	0.000	0.000
Eosinophils	0.000	0.000	0.000	0.000	0.000	0.000
Neutrophils	0.000	0.000	0.000	0.000	0.000	0.000
P-value	0.010	0.010	0.010	0.010	0.010	0.010
Spearman correlation coefficient (ρ)	0.302	0.306	0.313	0.309	0.304	0.305
RMSE	0.973	0.964	0.960	0.968	0.967	0.989

Note : Here are 12 representative samples with strong statistical significance ($P \leq 0.01$) and strong effect size ($\rho > 0.3$). For correlation analysis of 539 samples in the complete dataset, see the Github online dataset (https://github.com/Yiwen234/CXCL13-/blob/main/CIBERSORT-Results.xlsx)). The data in this table is used to generate the text figure 28.

Appendix S Supplementary Chart

Figure S1 : Spearman correlation heat map between 21 immune cell types revealed by CIBERSORT analysis (full version)

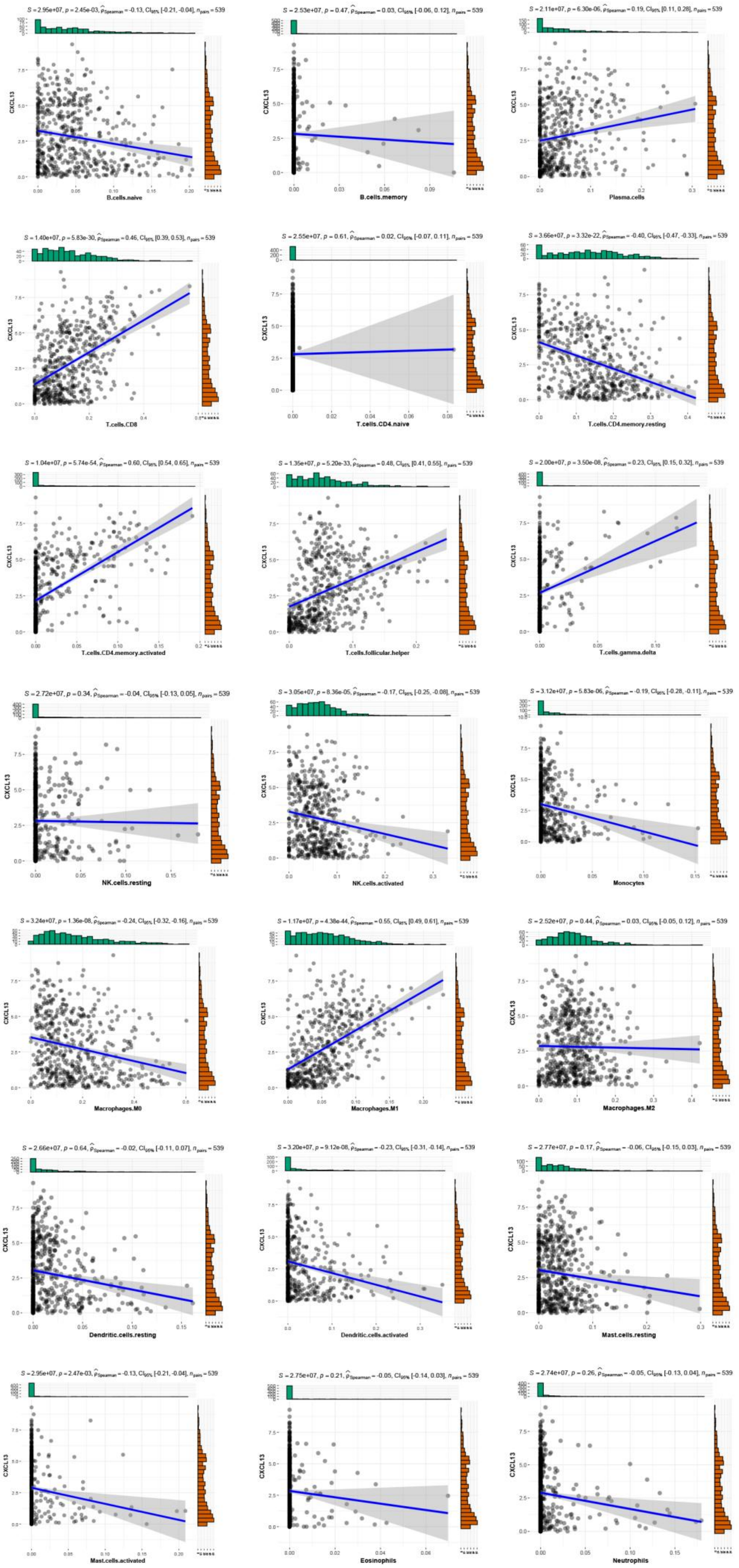
$S = 2.95e+07$, $p = 2.45e-03$, $\hat{\rho}_{Spearman} = -0.13$, $CI_{95\%}$ [-0.21, -0.04], $n_{pairs} = 539$
CXCL13
B.cells.naive
$S = 2.53e+07$, $p = 0.47$, $\hat{\rho}_{Spearman} = 0.03$, $CI_{95\%}$ [-0.06, 0.12], $n_{pairs} = 539$
CXCL13
B.cells.memory
$S = 2.11e+07$, $p = 6.30e-06$, $\hat{\rho}_{Spearman} = 0.19$, $CI_{95\%}$ [0.11, 0.28], $n_{pairs} = 539$
CXCL13
Plasma.cells
$S = 1.40e+07$, $p = 5.83e-30$, $\hat{\rho}_{Spearman} = 0.46$, $CI_{95\%}$ [0.39, 0.53], $n_{pairs} = 539$
CXCL13
T.cells.CD8
$S = 2.55e+07$, $p = 0.61$, $\hat{\rho}_{Spearman} = 0.02$, $CI_{95\%}$ [-0.07, 0.11], $n_{pairs} = 539$
CXCL13
T.cells.CD4.naive
$S = 3.66e+07$, $p = 3.32e-22$, $\hat{\rho}_{Spearman} = -0.40$, $CI_{95\%}$ [-0.47, -0.33], $n_{pairs} = 539$
CXCL13
T.cells.CD4.memory.resting
$S = 1.04e+07$, $p = 5.74e-54$, $\hat{\rho}_{Spearman} = 0.60$, $CI_{95\%}$ [0.54, 0.65], $n_{pairs} = 539$
CXCL13
T.cells.CD4.memory.activated
$S = 1.35e+07$, $p = 5.20e-33$, $\hat{\rho}_{Spearman} = 0.48$, $CI_{95\%}$ [0.41, 0.55], $n_{pairs} = 539$
CXCL13
T.cells.follicular.helper
$S = 2.00e+07$, $p = 3.50e-08$, $\hat{\rho}_{Spearman} = 0.23$, $CI_{95\%}$ [0.15, 0.32], $n_{pairs} = 539$
CXCL13
T.cells.gamma.delta
$S = 2.72e+07$, $p = 0.34$, $\hat{\rho}_{Spearman} = -0.04$, $CI_{95\%}$ [-0.13, 0.05], $n_{pairs} = 539$
CXCL13
NK.cells.resting
$S = 3.05e+07$, $p = 8.36e-05$, $\hat{\rho}_{Spearman} = -0.17$, $CI_{95\%}$ [-0.25, -0.08], $n_{pairs} = 539$
CXCL13
NK.cells.activated
$S = 3.12e+07$, $p = 5.83e-06$, $\hat{\rho}_{Spearman} = -0.19$, $CI_{95\%}$ [-0.28, -0.11], $n_{pairs} = 539$
CXCL13
Monocytes
$S = 3.24e+07$, $p = 1.36e-08$, $\hat{\rho}_{Spearman} = -0.24$, $CI_{95\%}$ [-0.32, -0.16], $n_{pairs} = 539$
CXCL13
Macrophages.M0
$S = 1.17e+07$, $p = 4.38e-44$, $\hat{\rho}_{Spearman} = 0.55$, $CI_{95\%}$ [0.49, 0.61], $n_{pairs} = 539$
CXCL13
Macrophages.M1
$S = 2.52e+07$, $p = 0.44$, $\hat{\rho}_{Spearman} = 0.03$, $CI_{95\%}$ [-0.05, 0.12], $n_{pairs} = 539$
CXCL13
Macrophages.M2
$S = 2.66e+07$, $p = 0.64$, $\hat{\rho}_{Spearman} = -0.02$, $CI_{95\%}$ [-0.11, 0.07], $n_{pairs} = 539$
CXCL13
Dendritic.cells.resting
$S = 3.20e+07$, $p = 9.12e-08$, $\hat{\rho}_{Spearman} = -0.23$, $CI_{95\%}$ [-0.31, -0.14], $n_{pairs} = 539$
CXCL13
Dendritic.cells.activated
$S = 2.77e+07$, $p = 0.17$, $\hat{\rho}_{Spearman} = -0.06$, $CI_{95\%}$ [-0.15, 0.03], $n_{pairs} = 539$
CXCL13
Mast.cells.resting
$S = 2.95e+07$, $p = 2.47e-03$, $\hat{\rho}_{Spearman} = -0.13$, $CI_{95\%}$ [-0.21, -0.04], $n_{pairs} = 539$
CXCL13
Mast.cells.activated
$S = 2.75e+07$, $p = 0.21$, $\hat{\rho}_{Spearman} = -0.05$, $CI_{95\%}$ [-0.14, 0.03], $n_{pairs} = 539$
CXCL13
Eosinophils
$S = 2.74e+07$, $p = 0.26$, $\hat{\rho}_{Spearman} = -0.05$, $CI_{95\%}$ [-0.13, 0.04], $n_{pairs} = 539$
CXCL13
Neutrophils

The heat map shows the pairwise Spearman correlation between the proportions of 21 tumor-infiltrating immune cells (TICs) subsets estimated based on the CIBERSORT algorithm in TCGA endometrial cancer (UCEC) samples. Only statistically significant correlations ($p < 0.05$) were shown in the figure, and the cells that did not reach the significant level were shown as white. The heat map system reveals the co-infiltration or mutual exclusion patterns between different immune cell subsets in the UCEC tumor microenvironment, providing a global perspective for understanding the interaction network between immune cells and its potential impact on tumor immunophenotype. The data in this table is used to generate the text figure 28.

Figure S2 : Single cell RNA sequencing data quality control : Correlation scatter diagram of total RNA molecules and mitochondrial gene percentage.

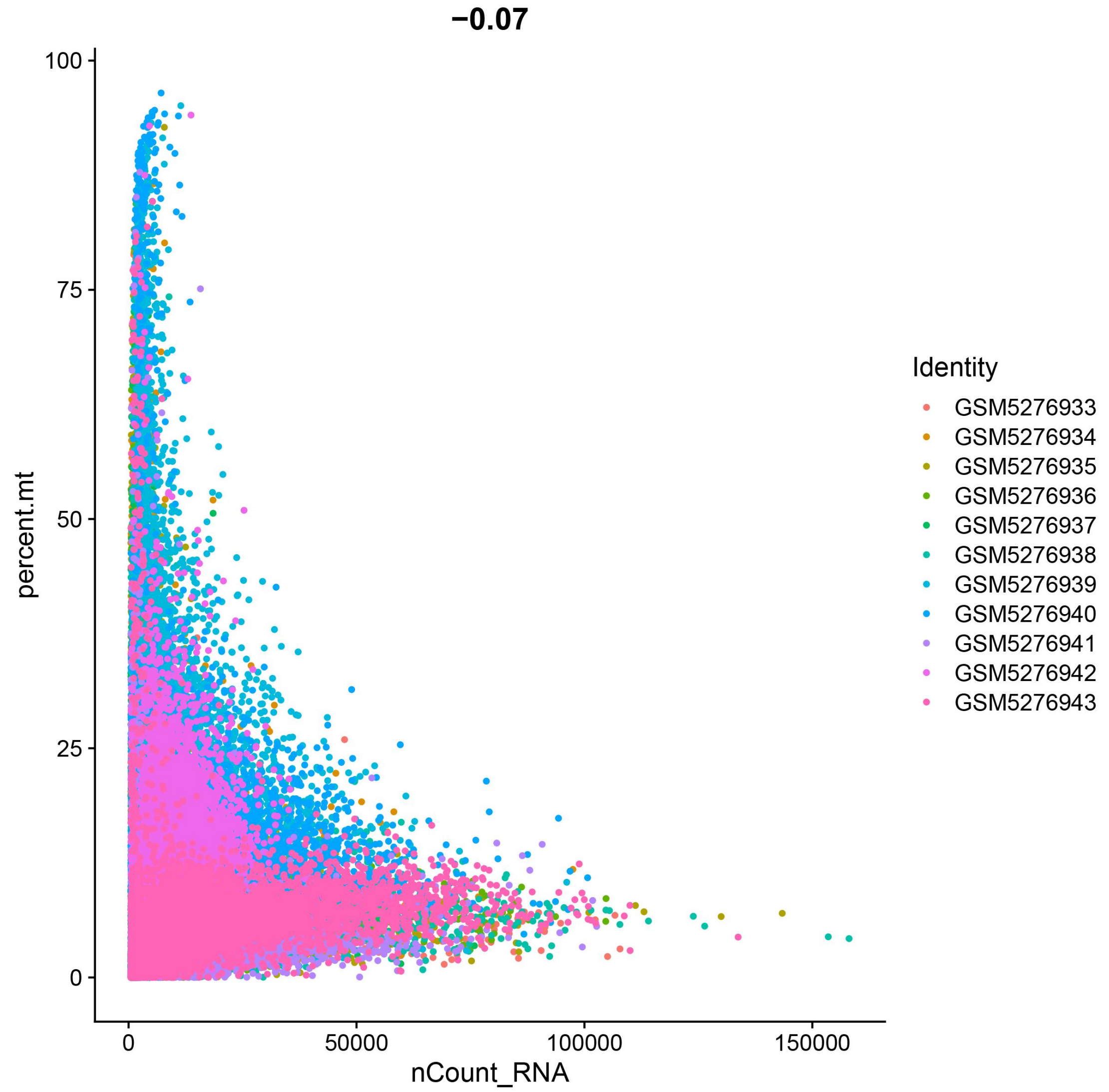

The figure shows the correlation between the number of total RNA molecules detected in each cell (nCount _ RNA) and the percentage of mitochondrial gene expression (percent.mt). Each point in the figure represents a cell, and the color corresponds to different sample sources (orig.ident, a total of 11 samples). By observing the relationship between the two, it can be evaluated whether the cells are abnormally highly expressed due to stress, apoptosis or technical errors, which can be used to screen low-quality cells. The cells in the upper right corner (high

nCount _ RNA and high percent.mt) were low-quality or stressed cells, which were eliminated in subsequent analysis.

Figure S3 : Single-cell RNA sequencing data quality control : Scatter plot of the correlation between the number of total RNA molecules and the number of detected genes

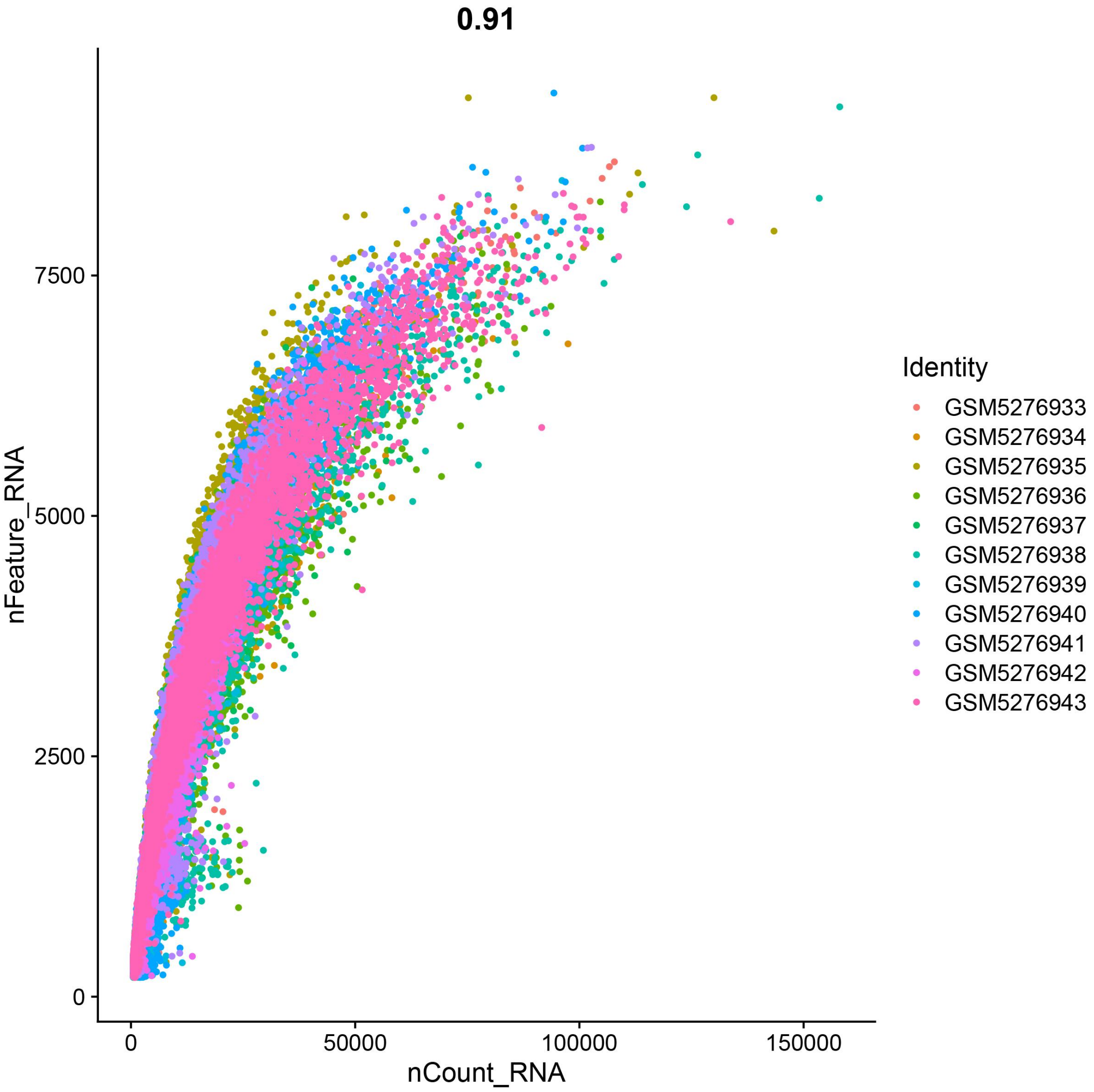

The figure shows the correlation between the number of total RNA molecules detected in each cell (nCount _ RNA, X axis) and the number of unique genes detected (nFeature _ RNA, Y axis). Each point in the figure represents a cell, and different colors correspond to 11 independent samples (orig.ident). In high-quality data, the two should show a good positive correlation. High-level correlation (point distribution along the diagonal) indicates good data quality ; outliers, especially those cells with abnormally high nCount _ RNA but disproportionate increase in nFeature _ RNA, may indicate that they are doublets or cells containing too much environmental RNA, which are eliminated in subsequent analysis.

Figure S4 : Violin plot of the first four key quality indicators of single cell RNA sequencing data quality control

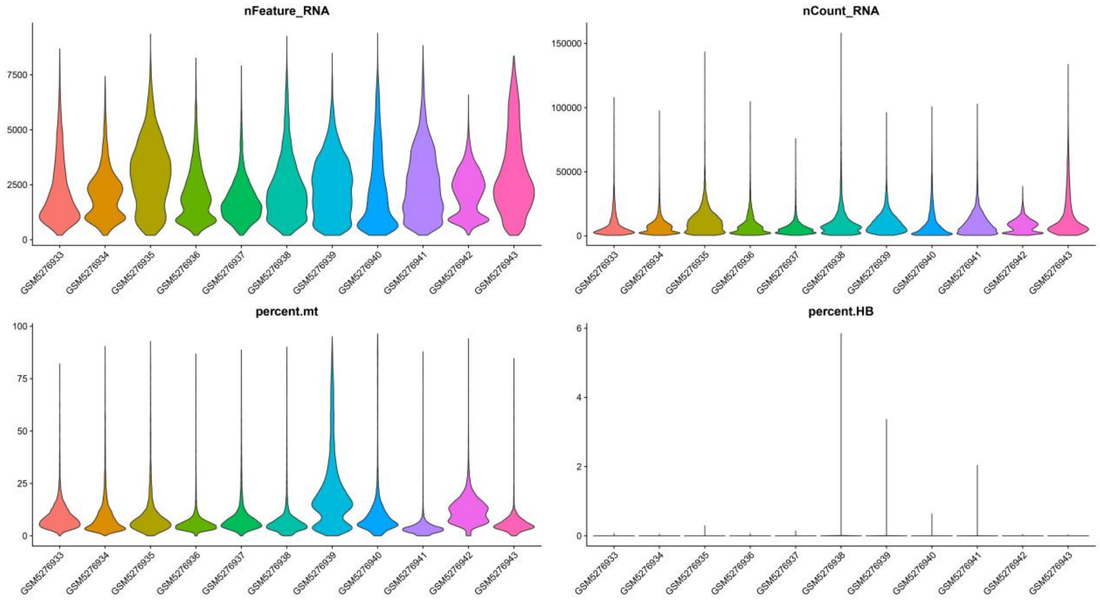

The combination diagram shows the distribution of four key quality indicators of 11 samples (GSM5276933-GSM5276943) before quality control. The number of genes detected (nFeature _ RNA) : reflects the abundance of genes expressed in each cell. Total RNA number (nCount _ RNA) : reflects the sequencing depth of each cell ; mitochondrial gene percentage (percent.mt) : reflecting the state of cell stress or apoptosis, high value may indicate cell damage ; percentage of hemoglobin gene (percent. HB) : Reflects the degree of red blood cell contamination.

Each violin plot shows the distribution of an indicator in all samples, which can be used to evaluate the consistency between samples and identify abnormal samples. The data before quality control is used to set the subsequent filtering threshold.

Figure S5 : Violin diagram of four key quality indicators after quality control of single-cell RNA sequencing data

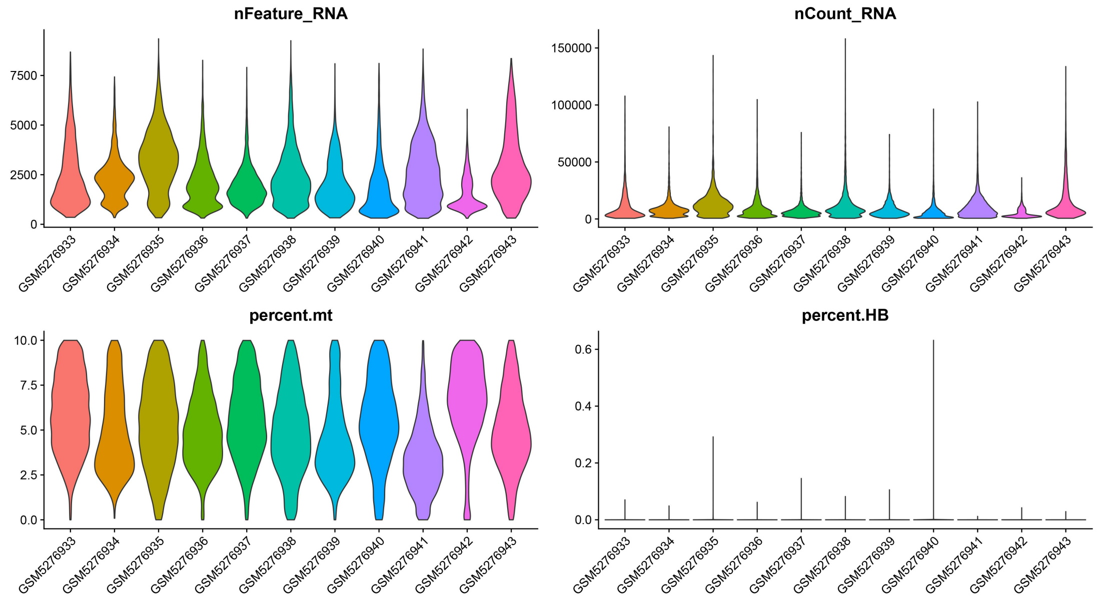

The figure shows the distribution of key quality indicators of retained cells in 11 samples after strict quality control threshold filtering. The filter threshold used is : the number of detected genes (nFeature _ RNA) is between 300 and 10000 ; the number of total RNA molecules (nCount _ RNA) was greater than 600 ; the percentage of mitochondrial gene (percent.mt) was less than 10 %. The percentage of hemoglobin gene (percent. HB) was less than 1 %. Compared with before quality control (Figure B4), the abnormal high or low values of all indicators have been effectively eliminated, and the distribution between samples is more uniform and concentrated, indicating that the data quality has been significantly improved, which can be used for subsequent dimensionality reduction, clustering and cell type annotation analysis.

Figure S6 : Comparison before and after batch effect correction (Harmony algorithm effect evaluation)

Figure S6.1 : Before batch effect correction : Cell type annotation and t-SNE dimensionality reduction visualization in single-cell transcriptome data

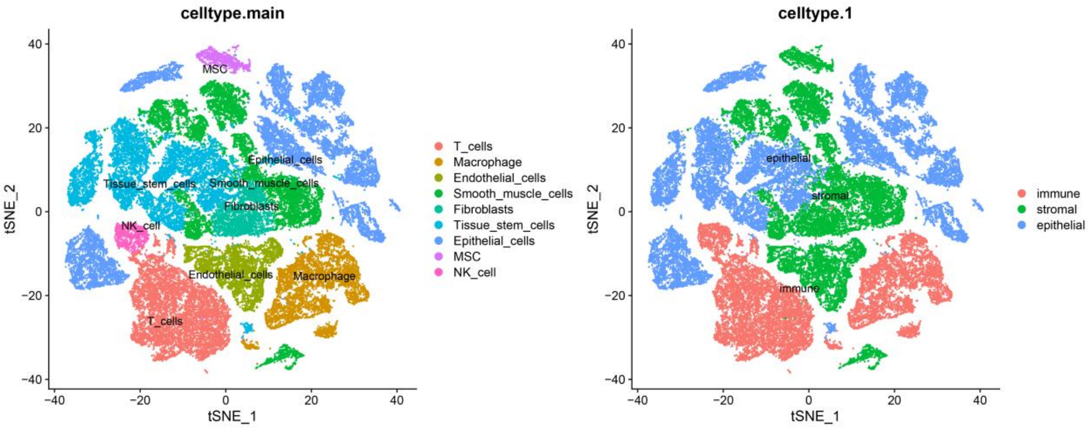

This figure shows the results of dimensionality reduction and cell clustering based on t-SNE (t-distributed Stochastic Neighbor Embedding) after quality control, normalization and batch correction of single-cell transcriptome data of endometrial cancer (UCEC). The left figure shows the distribution of cells in the t-SNE reduced-dimensional space. Different colors represent the nine main cell types initially annotated, including T cells, macrophages, endothelial cells, smooth muscle cells, fibroblasts, tissue stem cells, epithelial cells, mesenchymal stem cells (MSC) and natural killer cells (NK _ cells). The right figure shows that the same data set is divided into three categories according to the division of cell lineage : immune cells, stromal cells and epithelial cells. This figure intuitively reflects the spatial distribution and heterogeneity of different cell populations in the tumor microenvironment, and provides a spatial background for subsequent cell source and functional analysis of CXCL13.

Fig S6.2 : After batch effect correction : Single cell clustering and cell type distribution based on UMAP dimension reduction

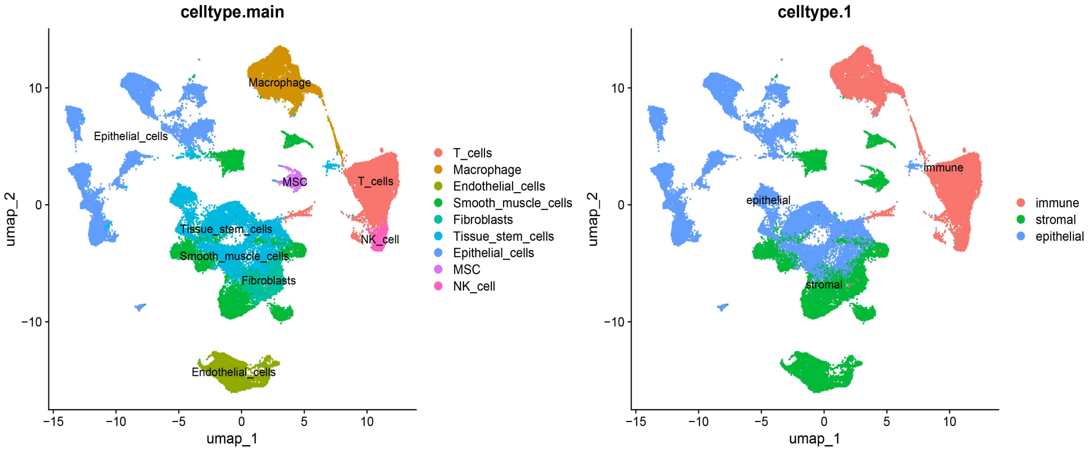

This map uses UMAP (Uniform Manifold Approximation and Projection) dimensionality reduction technology to visualize the single-cell transcriptome data of endometrial cancer. The above image shows the distribution of cells in the UMAP space, and the color represents the main cell types (celltype.main), including T cells, macrophages, epithelial cells, smooth muscle cells, fibroblasts, endothelial cells, tissue stem cells, MSC and NK cells, which visually shows the aggregation state of different cell populations in the dimensionality reduction space. The following figure further divides the cells into three major lineages : immune cells (immune), stromal cells (stromal) and epithelial cells (epithelial). The color corresponds to its lineage, revealing the overall distribution pattern of different cell components in the tumor microenvironment.

Appendix S Analysis Parameters and Software Versions

Software and Package Versions

- R version 4.5.1
- RStudio 2025 05.1 + 513
- Bioconductor version : 3.21.1 (BiocVersion package)

Single-cell analysis : Seurat (5.3.0), SingleCellExperiment (1.30.1), scran (1.36.0)

Differential expression : DESeq2 (1.48.2), edgeR (4.6.3), limma (3.64.1)

Enrichment analysis : clusterProfiler (4.16.0), DOSE (4.2.0)

Visualization : ggplot2 (3.5.2), ComplexHeatmap (2.24.1)

Data processing : dplyr (1.1.4), tidyverse (2.0.0)

Single cell quality control parameters

-Number of detected genes (nFeature _ RNA) : 300-10000

-Total RNA molecules (nCount _ RNA) : > 600

- Mitochondrial gene percentage (percent.mt) : < 10 %
- Percentage of hemoglobin gene (percent. HB) : < 1 %

-Double detection using DoubletFinder v2.0.6

Appendix S Key analysis code

S1 : Differential Expression Analysis (Take ImmuneScore Differential Analysis as an Example)

```
Library ( BiocManager )
library ( DESeq2 )
library ( tidyverse )
# TCGA difference analysis counts to do because it is the 01A group of patients do difference analysis so read 01A
counts _ 01A < -read.table ( ' counts01A.txt ', sep = ' \ t ', row.names = 1, check.names = F, stringsAsFactors = F, header = T )
# reads ESTIMATE _ result because it is grouped by immune score.
estimate < -read.table ( ' ESTIMATE _ result.txt ', sep = ' \ t ', row.names = 1, check.names = F, header = T )
# Organize grouping information
x < - ' ImmuneScore '
med < -as.numeric ( median ( estimate [, x ] ) ) # as.numeric as a numeric value
estimate < -as.data.frame ( t ( estimate ) ) # row-column conversion
identical ( colnames ( counts _ 01A ), colnames ( estimate ) )
# data.frame Create data frames
conditions = data.frame ( sample = colnames ( counts _ 01A ), # The first column name is sample
group = factor ( ifelse ( estimate [ x, ] > med, ' high ', ' low ' ), levels = c ( ' low ', ' high ' ) ) % > %
column _ to _ rownames ( ' sample ' )
# Disassemble the previous sentence length code
# conditions = data.frame ( sample = colnames ( counts _ 01A ).
# group = factor ( ifelse ( estimate [ x, ] > med, ' high ', ' low ' ), levels = c ( ' low ', ' high ' ) ) )
```

conditions < -column _ to _ rownames (conditions, ' sample ')

Difference Analysis Preparation
dds < -DESeqDataSetFromMatrix (
countData = counts _ 01A.
colData = conditions.
design = ~ group)

Start the difference analysis
dds < -DESeq (dds)
This sentence is very important.
ResultsNames (dds) # Remember whether the group is high / low or low / high.
Extraction results
res < -results (dds)
save (res, file = ' DEG _ ImmuneScore.Rda ')

heat map drawing # # #
DEG < -as.data.frame (res) # The second column log2 positive number is high expression, negative number is low expression and the last column padj regulates P value.
Read the expression profile
exp < -read.table (' tpms01A _ log2.txt ', sep = ' \ t ', row.names = 1, check.names = F, stringsAsFactors = F, header = T)
Add up and down information, the second column log2 is divided into high and low expression with 1 as the boundary, and the data that does not meet the standard is removed.
logFC _ cutoff < -2
type1 = (DEG$padj < 0.05)&(DEG$log2FoldChange < -logFC _ cutoff)
type2 = (DEG$padj < 0.05)&(DEG$log2FoldChange > logFC _ cutoff)
# Add a column
DEG $ change = ifelse (type1, ' DOWN ', ifelse (type2, ' UP ', ' NOT '))
table (DEG $ change)
Download pheatmap package
install.packages (' pheatmap ')
library (pheatmap)
Extraction of differential gene expression profiles
a < -filter (DEG, change = = ' UP ') # filter filter function
b < -filter (DEG, change = = ' DOWN ')
c < -rbind (a, b)
d < -rownames (c)
exp _ diff < -exp [d,]
Set grouping information
annotation _ col < -conditions
handles the order of the exp _ diff column
a < -filter (annotation _ col, group = = ' high ')
b < -filter (annotation _ col, group = = ' low ')

```
exp _ diff _ high < -exp _ diff [, rownames ( a ) ]
exp _ diff _ low < -exp _ diff [, rownames ( b ) ]
exp _ diff < -cbind ( exp _ diff _ high, exp _ diff _ low )
# Start drawing
color _ breaks < -seq ( 1.5, -1.5, length.out = 80 )
pheatmap ( exp _ diff.
Annotation _ col = annotation _ col.
scale = ' row '.
main = ' ImmuneScore ', # Add title
show _ rownames = F.
show _ colnames = F.
color = colorRampPalette ( c ( ' blue ', ' white ', ' red ' ) ) ( 80 ).
breaks = color _ breaks.
cluster _ cols = F, # column clustering
cluster _ rows = T.
fontsize = 10, # font size
fontsize _ row = 12.
fontsize _ col = 12 )
# Save the picture to adjust the size
dev.off ( ) # Close the drawing board Example 2
```

S2 : Survival Analysis (Take ImmuneScore Analysis as an Example)

```
library ( survival )
fitd < -survdiff ( Surv ( OS.time, OS ) ~ group, # associates OS.time, OS with group
data = surv.
na.action = na.exclude )
pValue < -1-pchisq ( fitd$chisq, length(fitd$n ) -1 )

# 2.2 Fitting survival curve
fit < -survfit ( Surv ( OS.time, OS ) ~ group, data = surv )
summary ( fit )
p.lab < -paste0 ( ' P ', ifelse ( pValue < 0.050, ' < 0.050 ', paste0 ( ' = ', round ( pValue, 3 ) ) ) ) ) ).

# install.packages ( ' survminer ' )
library ( survminer )
ggsurvplot ( fit.
data = surv.
pval = p.lab.
conf.int = TRUE, # Shows the confidence interval, the shadow part of the spread
risk.table = TRUE, #
break.time.by = 5, # x axis step is 5
legend.title = ' ImmuneScore ', # Title
surv.median.line = ' hv ', # Limits vertical and horizontal median survival
ylab = ' Survival probability ( % ) ', # Modify the y-axis label
```

```
xlab = ' Time ( Years ) ', # Modify the x-axis label
ncensor.plot = TRUE, # Show Censored Blocks
ncensor.plot.height = 0.25.
risk.table.y.text = FALSE )
ggsurvplot ( fit.
data = surv.
pval = p.lab.
conf.int = TRUE, # Shows the confidence interval, the shadow part of the spread
risk.table = TRUE, # Show risk table
risk.table.col = ' strata '.
palette = ' jco ', # color with jco, jama, lancet
legend.labs = c ( ' Low ', ' High ' ), # Legend
size = 1.
xlim = c ( 0, 15 ), # x axis length
break.time.by = 5, # x axis step is 5
legend.title = ' ImmuneScore ', # legend title
surv.median.line = ' hv ', # Limits vertical and horizontal median survival
ylab = ' Survival probability ( % ) ', # Modify the y-axis label
xlab = ' Time ( Years ) ', # Modify the x-axis label
ncensor.plot = TRUE, # Show Censored Blocks
ncensor.plot.height = 0.25.
risk.table.y.text = FALSE.
ggtheme = theme _ minimal ( ) + # Use theme _ minimal as the underlying theme
theme ( legend.title = element _ text ( size = 20 ) ) ) # Adjust the legend title to 14
dev.off ( )...
```

(The complete code can be obtained by https://github.com/Yiwen234/CXCL13-/blob/main/ code. R)

Appendix S Data Availability Statement

The data used in this study are from public databases :

TCGA UCEC data : https://portal.gdc.cancer.gov/

Single-cell dataset : GEO GSE173682
